# A Quantitative Evaluation of MIRU-VNTR Typing Against Whole-Genome Sequencing for Identifying *Mycobacterium tuberculosis* Transmission: A Prospective Observational Cohort Study

**DOI:** 10.1016/j.ebiom.2018.07.019

**Published:** 2018-08-01

**Authors:** David H. Wyllie, Jennifer A. Davidson, E. Grace Smith, Priti Rathod, Derrick W. Crook, Tim E.A. Peto, Esther Robinson, Tim Walker, Colin Campbell

**Affiliations:** a Nuffield Department of Medicine, University of Oxford, John Radcliffe Hospital, Headley Way, Oxford OX3 9DU, UK; b Public Health England Academic Collaborating Centre, John Radcliffe Hospital, Headley Way, Oxford OX3 9DU, UK; c The National Institute for Health Research, Health Protection Research Unit (NIHR HPRU) in Healthcare Associated Infections and Antimicrobial Resistance, University of Oxford, UK; d Tuberculosis Section, National Infection Service, Public Health England, 61 Colindale Avenue, London NW9 5EQ, UK; e Public Health England National Regional Mycobacteriology Laboratory North and Midlands, Heartlands Hospital, Birmingham BS9 5SS

**Keywords:** *Mycobacterium tuberculosis*, MIRU-VNTR, Single nucleotide variation, Outbreak investigation, Molecular epidemiology, Research in context

## Abstract

**Background:**

Mycobacterial Interspersed Repetitive Unit-Variable Number Tandem Repeat (MIRU-VNTR) typing is widely used in high-income countries to determine *Mycobacterium tuberculosis* relatedness. Whole-genome sequencing (WGS) is known to deliver greater specificity, but no quantitative prospective comparison has yet been undertaken.

**Methods:**

We studied isolates from the English Midlands, sampled consecutively between 1 January 2012 and 31 December 2015. In addition to routinely performed MIRU-VNTR typing, DNA was extracted from liquid cultures and sequenced using Illumina technology. Demographic and epidemiological data for the relevant patients were extracted from the Enhanced Tuberculosis Surveillance system run by Public Health England. Closely related samples, defined using a threshold of five single nucleotide variants (SNVs), were compared to samples with identical MIRU-VNTR profiles, to samples from individuals with shared epidemiological risk factors, and to those with both characteristics.

**Findings:**

1999 patients were identified for whom at least one *M. tuberculosis* isolate had been MIRU-VNTR typed and sequenced. Comparing epidemiological risk factors with close genetic relatedness, only co-residence had a positive predictive value of over 5%. Excluding co-resident individuals, 18.6% of patients with identical MIRU-VNTR profiles were within 5 SNVs. Where patients also shared social risk factors and ethnic group, this rose to 48%. Only 8% of MIRU-VNTR linked pairs in lineage 1 were within 5 SNV, compared to 31% in lineage 4.

**Interpretation:**

In the setting studied, this molecular epidemiological study shows MIRU-VNTR typing and epidemiological risk factors are poorly predictive of close genomic relatedness, assessed by SNV. MIRU-VNTR performance varies markedly by lineage.

**Funding:**

Public Health England, Health Innovation Challenge Fund, NIHR Health Protection Research Unit Oxford, NIHR Oxford Biomedical Research Centre.

## Highlights

• *M. tuberculosis* relatedness was measured by MIRU-VNTR typing and single nucleotide variants (SNV).
• In consecutive isolates in England, only 20% of samples with identical MIRU-VNTR profiles were within 5 SNVs.
• MIRU-VNTR is more predictive of close relatedness in lineage 4 (Euro-American) lineages than in other lineages.

## Evidence Before This Study

We searched Pubmed using the search terms ‘whole genome sequencing’ and ‘MIRU-VNTR’ and ‘tuberculosis’ for English language articles published up to December 21st, 2017. Multiple studies have shown that most pairwise genomic comparisons will be within five SNVs when direct transmission has occurred from one individual to another. Both outbreak studies and population studies have demonstrated how whole-genome sequencing generates smaller clusters than MIRU-VNTR typing, and how sequence data allows for differentiation of isolates within a cluster. However, no systematic comparison of MIRU-VNTR typing vs. WGS has however been published. The degree to which WGS provides more specific results, and the degree to which it is likely to be more cost effective, therefore remains uncertain.

## Added Value Of This Study

This study seeks to quantify the predictive value of identical MIRU-VNTR profiles, and of overlapping demographic and epidemiological data, for close genomic relatedness in a cosmopolitan setting. Importantly, it demonstrates that in our setting MIRU-VNTR-based clustering predicts genomic relatedness differently depending on *M. tuberculosis* lineage. This is compatible with previous reports of poor discrimination by MIRU-VNTR in lineage 2 (Beijing), but is not restricted to lineage 2, and is likely to be generalizable to other settings. Our results provide an explanation as to why MIRU-VNTR typing was not cost effective when implemented in England, and indicate that WGS may perform substantially better.

## Implications of All the Available Evidence

Whilst it is generally accepted that WGS provides more informative results than MIRU-VNTR typing, the latter is still practiced widely under the belief that it remains a helpful tool for public health investigations. This study shows that whilst differing MIRU-VNTR profiles help exclude close genomic relatedness, matching profiles rarely predict such relatedness. Having quantified its predictive value at a population level, this study should hasten the transition from MIRU-VNTR typing to WGS in other settings similar to ours.

## 1 Introduction

In 2016 there were 5664 notified cases of tuberculosis in the England, with an incidence of 10.2 per 100,000 population [1]. Despite a steady fall in incidence since its peak early this decade, this remains the highest rate in western Europe, outside of the Iberian peninsula [2]. This decline has occurred across almost all population groups with only a third due to decreases in the numbers of migrants from high TB burden countries. Despite decreases in TB rates, domestic transmission is still likely to be contributing to current case loads [3].

Rapid detection of *Mycobacterium tuberculosis* transmission should offer enhanced opportunities for disease control [4, 5]. In England, as in many high-income countries, tuberculosis transmission has been identified with the help of Mycobacterial Interspersed Repetitive Unit-Variable Number Tandem Repeat (MIRU-VNTR) typing, which clusters cultured isolates on the basis of their molecular fingerprints [6, 7]. A recent post-deployment evaluation of the MIRU-VNTR-based surveillance programme in England has however questioned the cost-effectiveness of this approach [8].

Since 2015, Public Health England has been undertaking a phased introduction of routine whole genome sequencing (WGS) for all mycobacterial cultures [9]. This has meant the relatedness of isolates could be simultaneously compared using both single nucleotide variants (SNV) and by MIRU-VNTR typing, and has provided a novel opportunity to compare the added value of whole genome sequencing ([[10], [11], [12], [13], [14], [15]];Table 1) in an unselected population, at scale. This approach contrasts with recent studies in which samples from diverse geographic locations were selected by lineage, with selected subsets being characterised by both SNV and MIRU-VNTR [16, 17]. Analysis of unselected samples, as practiced here, can be used to investigate reports that MIRU-VNTR typing differentiates parts of Lineage 2 [16] [18], as other lineages [19], poorly.

**Table 1:** Previous studies including both MIRU-VNTR and SNV analysis of *M. tuberculosis*.

Samples	Comment	Reference
36 archived Manila strain isolates	SNV analysis revealed variation not demonstrated by MIRU-VNTR.	10
390 retrospective isolates from the English Midlands	Genetic heterogeneity within MIRU-VNTR clusters demonstrated. 5 and 12 SNV proposed as potential cut offs for epidemiological relatedness.	11
199 epidemiologically linked cases sequenced retrospectively	Relationship with MIRU-VNTR profile was not addressed	37
36 isolates from an outbreak	SNV analysis revealed variation not demonstrated by MIRU-VNTR.	38
50 cases from an outbreak	SNV analysis revealed variation not demonstrated by MIRU-VNTR.	12
1000 isolate sample of 2248. Representative of Russian population studied, plus 28 diverse sequences	Relationship with MIRU-VNTR profile was not addressed.	39
	Multiple sub-lineages observed within Lineage 4 (Euro-American).	
69 cases from an outbreak defined by a SNV	SNV analysis revealed variation not demonstrated by MIRU-VNTR.	13
86 cases from an outbreak	SNV analysis revealed variation not demonstrated by MIRU-VNTR.	14
90 cases belonging to 35 MIRU-VNTR clusters	MIRU-VNTR performance overestimated transmission particularly in immigrants infected with closely related strains	15
4987 lineage 2 samples representative of global diversity studied by MIRU-VNTR	110 specimen sample was sequenced by next-generation sequencing. MIRU-VNTR poorly defined some branches of the lineage 2 phylogeny	16
Paired isolates from 390 patient selected due to possible emergence of drug resistance	SNV analysis as well as MIRU-VNTR profiling used to confirm or exclude re-infection	40

Here we estimate what proportion of *M. tuberculosis* isolates from a cosmopolitan area of central England that are linked by MIRU-VNTR typing, or have associated epidemiological risk factors, are closely genomically related. In this work, we use SNV as a metric of close genetic similarity; although other kinds of variation, including insertions and deletions (indels) exist [20], here we chose to use SNV, for which cutoffs reflecting close genetic relatedness have been derived a in a range of populations [21], and for which the clock rate has been heavily studied [21], including external calibration against historical events [16].

## 2 Methods

### 2.1 Samples Studied for Comparison of MIRU-VNTR With SNVs

Consecutive *M. tuberculosis* isolates from the Public Health England Centre for Regional Mycobacteriology Laboratory, Birmingham between 1 January 2012 and 31 December 2015 were included in the study. This corresponds to the period when both MIRU-VNTR and SNV analysis were both performed. This laboratory serves a large catchment of approximately 12 million persons in the English Midlands, a region which includes high, medium (40–150 cases per 100,000 population), and low TB incidence areas. After exclusions, described in Results, 1999 isolates each isolated from a single patient, were studied.

### 2.2 Identification and MIRU-VNTR Typing

Clinical samples were grown in Mycobacterial Growth Indicator tubes (MGIT) (Becton Dickinson, New Jersey, USA), and *M. tuberculosis* was identified using Ziehl-Neelsen staining, followed by nucleic acid amplification and hybridisation using Genotype Mycobacterium CM hybridisation tests (Hain LifeScience, Nehren, Germany). 24-locus MIRU-VNTR typing [6, 22] was performed on the first isolate from each patient in each calendar year using non-denaturing HPLC (WAVE microbial analysis system) as described [23]. This assay demonstrated complete concordance with gel based fragment size analysis during the validation study in 2004 [23]. A detailed verification study, performed in 2014, indicated that assay performance had not changed substantially relative to the validation study (Supplementary Data 1). Throughout use, the assay was subject to internal and external quality control.

### 2.3 Laboratory and Bioinformatic Processing

This was carried out as described [11]. Nucleic acid was extracted from 1·7 ml of MGIT culture as described [9]. Illumina 150 bp paired end DNA libraries were made using Nextera XT version 2 chemistry kits and sequenced on MiSeq instruments (Illumina). Reads were mapped to the H37Rv v2 reference genome (Genbank: NC000962.2) using Stampy [24], and aligned to Bam files parsed with Samtools mPileup [25], with further filtering performed based on the base and alignment quality (q30 and Q30 cutoffs, respectively). Mean depth of high-quality mapping per genome was typically between 50 and 100. Bases supported only by low confidence base calls were recorded as uncertain (‘N’), as were positions with >10% minor variant frequencies, and all calls at the genomic positions included in Supplementary Data 2, since these regions were repetitive (as identified by self-self blastn analysis) or were found to commonly contain low-confidence mapping (*rrl*, *rrs*, *rpoC* and *Rv2082* loci) [26]. Such uncertain bases were ignored in pairwise single nucleotide variation (SNV) computations reported in this work.

Thus, we define a SNV as existing between two sequences at a particular base when the minor variant frequency is <10% in both sequences, and the major variant differs between the two sequences, and the base is not in a region known to be repetitive or contain low confidence mapping.

### 2.4 Metrics of Relatedness

We used pairwise SNV distances between isolates as a metric of close genetic relatedness, considering isolates closely genetically related when their pairwise SNV distance was less a particular SNV threshold. In this analysis we did not exclude resistance loci, because acquired resistance is very rare in the setting studied [3]. For the main analysis, 5 SNV was used as the threshold, but other thresholds were considered in sensitivity analyses.

Lineage assignation was performed using ancestral SNVs, as described [27]. Relatedness between samples was determined by comparing the number of mismatching positions between loci using BugMat [28]. Relatedness between MIRU-VNTR profiles compared the total number of loci different between isolate pairs. For example, for a one-locus typing scheme, if isolate 1 had 3 repeats, and isolate 2 had 5 repeats, we coded this as one difference. We used this policy because of evidence that single evolutionary events can lead to changes in repeat length of more than one unit [18, 29]. If the MIRU-VNTR repeat number at the locus could not be determined in isolate 1, and isolate 2 had two repeats, we counted this as no difference.

### 2.5 Collection and Collation of Patient Data

Demographic data (sex, age, ethnic group and residence), and social risk factor data (current or history of imprisonment, drug misuse, alcohol misuse or homelessness) were obtained from the Enhanced Tuberculosis Surveillance system. Co-residence was defined as having the same first line of address and postcode.

### 2.6 Statistical Analyses

We considered a series of categorical variables as predictors of close genomic relatedness in logistic regression analyses. Additionally, for some variables, we constructed composite categorical variables reflecting whether more than one risk factor was present. For each given SNV threshold, we estimated odds ratios for close genomic relatedness using logistic regression. Separately, we modelled the relationship between SNV variation (s) (outcome), *Mycobacterium tuberculosis* lineage (l, a discrete variable) and n, the number of MIRU-VNTR repeat number differences observed, as defined above. We modelled

$$E\left(s\right)\sim n+l+n^{*}l$$

thus allowing estimation of both lineage-specific variation in the absence of any variation in MIRU-VNTR types, and how SNV increased with increasing MIRU-VNTR differences. We used quantile regression (R quantreg package) for the main analysis as homoscedascity assumptions were violated. All analyses used R 3.3.1 for Windows.

### 2.7 Ethical Framework

Public health action taken as a result of notification and surveillance is one of the Public Health England's key roles as stated in the Health and Social Care Act 2012 and subsequent Government directives which provide the mandate and legislative basis to undertake necessary follow-up. Part of this follow-up is identification of epidemiological and molecular links between cases. This work is part of service development carried out under this framework, and as such explicit ethical approval is unnecessary.

### 2.8 Funding Source

This study is supported by the Health Innovation Challenge Fund (a parallel funding partnership between the Wellcome Trust [WT098615/Z/12/Z] and the Department of Health [grant HICF-T5–358]) and NIHR Oxford Biomedical Research Centre. Professor Derrick Crook is affiliated to the National Institute for Health Research Health Protection Research Unit (NIHR HPRU) in Healthcare Associated Infections and Antimicrobial Resistance at University of Oxford in partnership with Public Health England. Professor Crook is based at University of Oxford. The views expressed are those of the author(s) and not necessarily those of the NHS, the NIHR, the Department of Health or Public Health England. The sponsors of the study had no role in study design, data collection, data analysis, data interpretation, or writing of the report. The corresponding author had full access to all the data in the study and had final responsibility for the decision to submit for publication.

## 3 Results

### 3.1 Isolates Studied

**Fig. 1:**
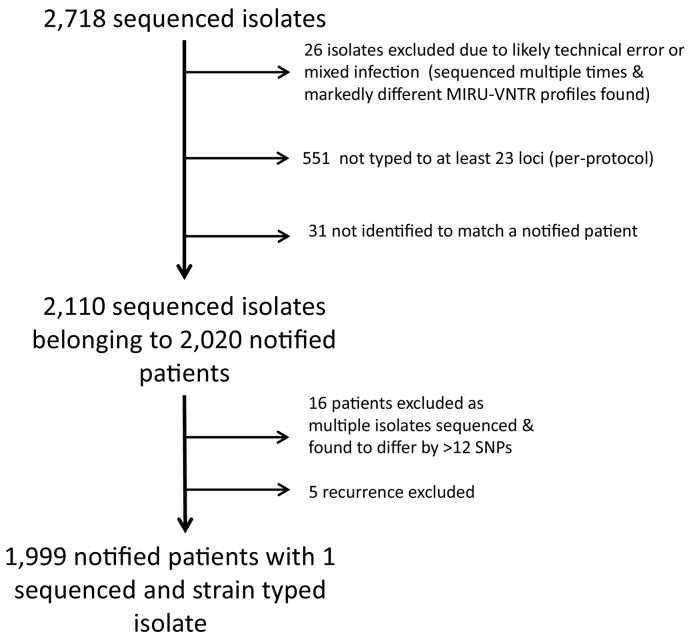
Flowchart showing the samples studied. Flowchart showing the samples studied.

We studied all *M. tuberculosis* isolates consecutively grown in, or referred to, the Public Health England Mycobacterial reference centre for the English Midlands between 2012 and 2015 (*n* = 2718) (Fig. 1). We excluded 551 isolates because MIRU-VNTR typing had already been performed on a previous isolate within that calendar year, as was protocol at the time. We also excluded 57 isolates because of concerns about laboratory processing (Fig. 1), including different MIRU-VTNR types on repeated analysis, suggestive or either mixed infection or technical error. The remaining 2110 isolates came from 2020 discrete patients. A further 16 isolates were excluded because multiple isolates from the same individual were separated by >12 single nucleotide variants (SNVs) (suggestive of either technical error or infection with multiple strains), along with five recurrent cases of *M. tuberculosis* infection, leaving 1999 isolates each derived from a different patient.

There were more male than female patients (1176, 58%). 1155 (58%) were aged between 15 and 44 years old. 1325 patients (66%) were born outside the UK and 1437 (71%) were of non-White ethnicity (Table 2). *M. tuberculosis* lineage 4 (Euro-American) was the most commonly isolated lineage (*n* = 954, 48%) with lineages 1, 2, and 3 also commonly represented (176 (9%), 137 (7%), 704 (35%) isolates respectively) (Table 3). *M. tuberculosis* lineage was associated with country of birth, with lineage 3 being most common in individuals born in India or Pakistan (Table 3).

**Table 2:** Details of Samples studied.

Category	Property	Number of samples
Number of social risk factors (homelessness, prison, alcohol use, drug use)	0	1761
	1	136
	2	51
	3	26
	4	3
	Not available	22
Gender	Female	801
	Male	1176
	Not available	22
	0–14	45
	15–44	1155
	45–64	442
	65+	335
Age group	Not available	22
Year sample taken	2007	1
	2010	1
	2011	5
	2012	355
	2013	584
	2014	507
	2015	524
	Not available	22
PHE Region of patient's residence	London	6
	Midlands & East of England	1721
	North of England	243
	South of England	3
	Not available	26
Self-declared ethnic group	Bangladeshi	31
	Black-African	267
	Black-Caribbean	57
	Black-Other	14
	Chinese	29
	Indian	564
	Mixed / Other	143
	Pakistani	332
	White	508
	Not available	54
UK Born	Non-UK Born	1325
	UK Born	592
	Not available	82

**Table 3:** Lineage of isolates studied.

	Lineage					
Place of birth	1	2	3	4	Other	Total
UNITED KINGDOM	19 (3·2%)	33 (5·5%)	136 (23%)	391 (66%)	13 (2·1%)	592 (100%)
INDIA	81 (18%)	18 (4·0%)	246 (55%)	102 (23%)	1 (0·2%)	448 (100%)
PAKISTAN	16 (6·3%)	6 (2·3%)	178 (71%)	51 (20%)	1 (0·4%)	252 (100%)
SOMALIA	8 (15%)	2 (3·8%)	24 (45%)	18 (33%)	1 (1·9%)	53 (100%)
ZIMBABWE	3 (6·0%)	7 (14%)	1 (2·0%)	37 (76%)	1 (2·0%)	49 (100%)
ERITREA	3 (6·5%)	2 (4·3%)	16 (35%)	25 (54%)	0 (0·0%)	46 (100%)
POLAND	0 (0·0%)	1 (2·8%)	1 (2·8%)	34 (94%)	0 (0·0%)	36 (100%)
ROMANIA	0 (0·0%)	0 (0·0%)	0 (0·0%)	28 (100%)	0 (0·0%)	28 (100%)
LITHUANIA	0 (0·0%)	8 (33%)	0 (0·0%)	16 (66%)	0 (0·0%)	24 (100%)
Other	36 (9·5%)	58 (15%)	66 (18%)	208 (55%)	9 (2·4%)	377 (100%)
Not known	10 (10%)	2 (2·1%)	36 (38%)	44 (49%)	2 (2·1%)	94 (100%)
Total	176	137	704	954	28	1999

### 3.2 Epidemiological Risk Factors and the Prediction of Close Relatedness

**Fig. 2:**
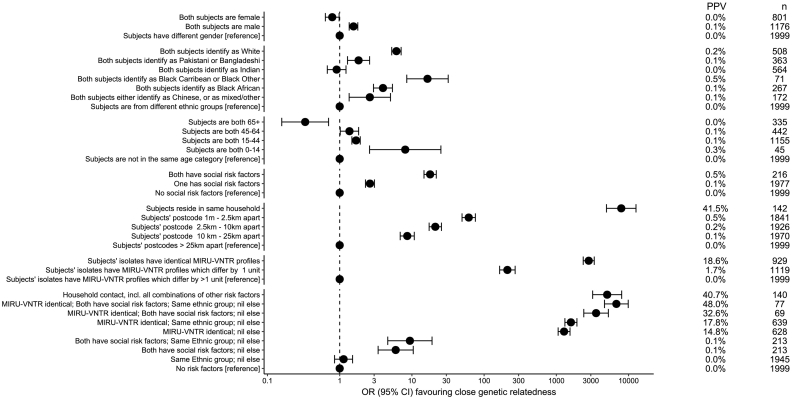
Close genetic relatedness given shared epidemiological risk factors or MIRU-VNTR profiles. The odds ratio favouring closely related isolates (defined by having five or fewer single nucleotide variants between them) when isolate pairs share a series of epidemiological properties or MIRU-VNTR profiles, relative to when they do not. PPV denotes positive predictive values. n refers to the number of subjects having the property described. For example, there were 801 female subjects.

Using pairwise SNV distances within 5 SNVs between isolates to define genomic relatedness, we determined how various shared epidemiological data altered the odds of relatedness. Fig. 2 shows estimated odds ratios of close genomic relatedness, in the presence, relative to the absence, of a series of risk factors. The proportion of paired isolates that are closely genomically related, given a particular risk factor, was also calculated. This represents the positive predictive value (PPV) of each risk factor. Other SNV thresholds were applied in sensitivity analyses (Supplementary Material 3, Fig. S1-S4), with similar results.

Predictably, residence at the same address was most strongly associated with close genomic relatedness (OR 8000, 95% CI 5000, 13,000). This corresponds to a PPV of 42%, indicating the majority of co-resident cases in this series were not closely genomically related, something discussed below. However, it was rare for two patients to share an address, with only 85 isolates derived from such settings. Being resident close to another case was also associated with an increased risk of close genomic relatedness, indicating that transmission within a restricted geographical area occurred.

This geographically restricted transmission allows a way of checking whether the 5 SNV cutoff used in our main analysis, which is informed by external evidence [21], is appropriate for this dataset. As pairwise SNV distances increase, we expect the geographic structuring of the data to become less evident as pairwise SNV distances become less compatible with transmission events. We illustrate this in Web extra Fig. S5. Only if close genetic relatedness is considered to occur with pairwise SNV distances of five or less is living close to another TB case is positively associated with close genetic relatedness.

Other risk factors studied included sharing a self-identified ethnic group with another patient or being in a similar age bracket. Both were weakly associated with genomic relatedness (estimated odds ratios of 10 or less), with the highest risk of close genomic relatedness for an ethnic group seen for the smallest ethnic group studied (those identifying as Black Caribbean or Black Other; *n* = 71; OR 16, 95% CI 8, 32). Similarly, there was a modest increase in the odds of close genomic relatedness where two isolates were from individuals with social risk factors (current or history of imprisonment, drug misuse, alcohol misuse or homelessness) (OR 9, 95% CI 4, 16). In all these cases however, the PPV was <1%.

### 3.3 MIRU-VNTR Profiles as Predictors of Close Relatedness

Having identical MIRU-VNTR profiles conferred an odds ratio of close genomic relatedness of 2800 (95% CI 2200, 3400) on paired isolates, compared with paired isolates with different MIRU-VNTR profiles, with an associated 18.6% PPV (Fig. 2). With 1 locus discordant, the corresponding odds ratio and PPV were much lower (OR 210, 95% CI 160,270; PPV 1·7%).

To understand how MIRU-VNTR profile and epidemiological data can complement each other in the identification of close relatedness, we assessed combinations of the presence of identical MIRU-VNTR profiles, social risk factors, and shared ethnicity, all factors which are significantly associated with close relatedness individually (Fig. 2; data plotted is in Supp Data 2.). Excluding individuals who were resident at the same address, identical MIRU-VNTR profile was more predictive of close relatedness when shared risk factors were present, but for all the combinations studied the PPV remained low (15%, 18%, 33%, 48% with no shared risk factors, same ethnic group but no social risk factors, shared social risk factors but different ethnic group, and both shared ethnic group and social risk factors, respectively).

### 3.4 SNV - MIRU-VNTR Relationships Vary by Lineage

**Fig. 3:**
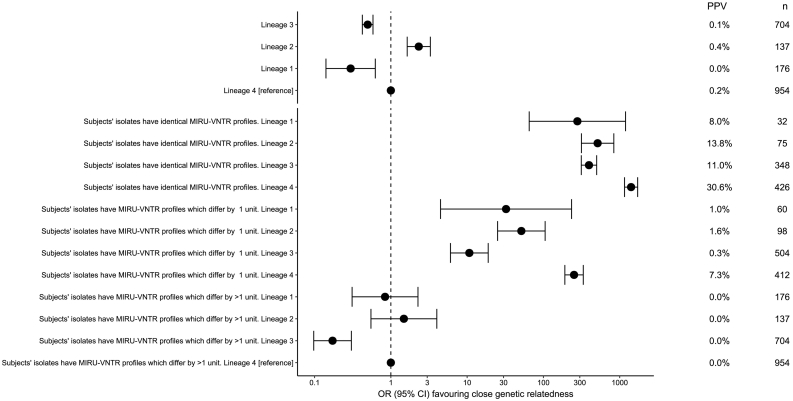
Close genetic relatedness given shared lineage and MIRU-VNTR profiles. The odds ratio favouring closely related isolates (defined by having five or fewer single nucleotide variants between them) when isolate pairs share a particular lineage (relative to lineage 4), or having identical or similar MIRU-VNTR profiles. PPV denotes positive predictive values. n refers to the number of subjects having the property described. For example, there were 954 subjects of lineage 4.

**Fig. 4:**
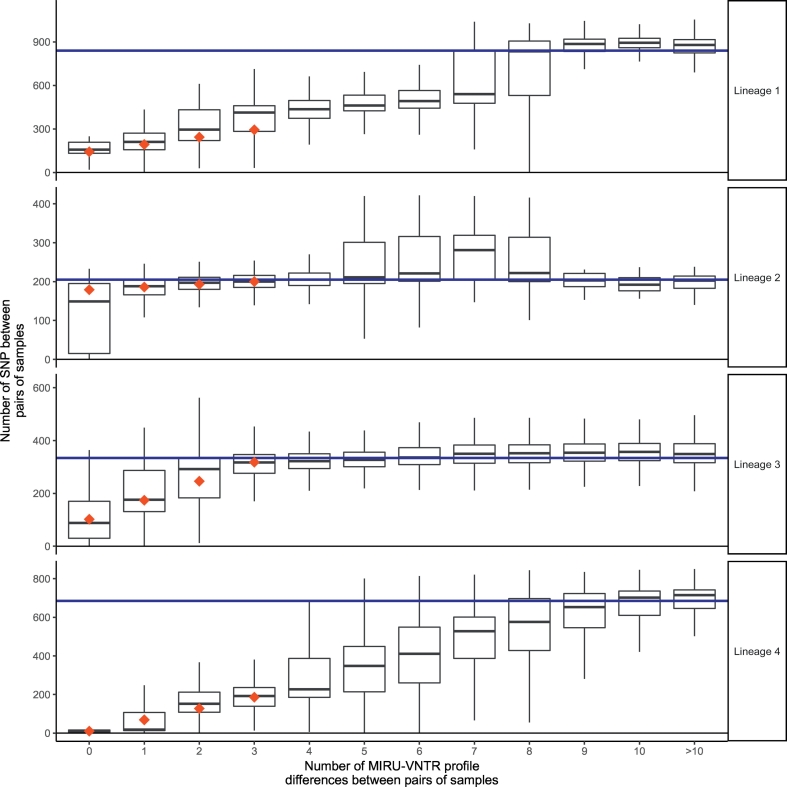
The relationship between MIRU-VNTR profile variation and SNV variation, stratified by lineage. The relationship between MIRU-VNTR profile variation and SNV variation, stratified by lineage. The x-axis shown the number of MIRU-VNTR loci differing between pairs of isolates. For example, if a sample had a MIRU-VNTR profile of 131, and another 111, one locus differs, which counts as a 1 MIRU-VNTR profile repeat number change. The y-axis shows the median number of SNV in each of a large number of pairs examined. The blue line reflects the median pairwise distance within all sampled isolates of each lineage. Red dots are fitted median values from a multivariable quantile regression model relating SNV (dependent variable) to lineage, MIRU-VNTR profile difference between 0 and 5 loci, inclusive, and their interaction.

While MIRU-VNTR profiles predict close genetic relatedness (defined by SNVs) better than most social risk factors (Fig. 2), we observed that the PPV differs markedly by *M. tuberculosis* lineage (Fig. 3). For lineages 1, 2, 3 and 4, which together account for 1977/1999 (99%) of the isolates studied, we compared pairwise comparisons within each lineage by MIRU-VNTR similarity (Fig. 4). For lineages 1 and 4, pairwise SNV distances increased over the range 0 to 8 MIRU-VNTR unit differences, until at higher MIRU-VNTR distances the pairwise distances approximated the within-lineage median pairwise SNV distance (Fig. 4). For lineages 2 and 3 the median was reached by 3 MIRU-VNTR differences. Overall there was less variation between paired isolates within lineages 2 and 3 (median pairwise distances 205 and 334, respectively) compared to paired isolates within lineages 1 and 4 (median pairwise distances 840, and 685). However, for paired isolates differing by between zero and 4 MIRU-VNTR loci, the least variation was seen within lineage 4.

To quantify how the relationship between MIRU-VNTR and SNVs differed by lineage, we modelled SNV distances between paired isolates, assuming (as is suggested from the observations, Fig. 4) a linear relationship with MIRU-VNTR profile distances over the range of 0–3 MIRU-VNTR locus differences (Fig. 4; Supp. Data 3). Over this range, we modelled single nucleotide variation as a function of numbers of differing MIRU-VNTR loci. We used quantile regression, which models median SNV, because homoplasy can create very large SNV distances between organisms with identical MIRU-VNTR profiles; such occurrences have high influence of ordinary least squares based regression.

In Fig. 4, red dots show fitted medians, which closely approximate the observed medians; the model indicates that for lineage 4 isolates, among pairs with identical MIRU-VNTR profiles, there was a median of 10 ± 0·4 SNV (median ± standard error). For paired isolates with identical MIRU-VNTR profiles in lineages 1, 2, and 3, SNV distances were 122 ± 21, 159 ± 3, and 82 ± 3 (median ± standard error), respectively. According to current estimates of *M. tuberculosis* clock rates, these correspond to divergence from a common ancestor up to 125, 150, and 75 years of evolution, respectively, compared to about 10 years for lineage 4 [21].

For each MIRU-VNTR locus difference in lineage 4, there was a median (SE) increase of 59 ± 0·6 SNV. For lineage 1, a similar increase in SNV with increasing MIRU-VNTR differences was observed to that in lineage 4 (median 50.7 ± 8.3, het*. p* = 0·32), whereas for lineages 2 and 3 the relationship was very different from lineage 4 (7.0 ± 8.3, 71.7 ± 0.7., respectively; het. *p* < 10^−20^ for both comparisons); for paired isolates in lineage 2, SNVs were not significantly associated with MIRU-VNTR distance. Thus, in the population studied, the performance of MIRU-VNTR profiles in defining evolutionarily related groups differed between lineage 4 (Euro-American) isolates, and lineages 1, 2 and 3.

### 3.5 SNV - MIRU-VNTR Relationships Vary Within Lineage 4

**Fig. 5:**
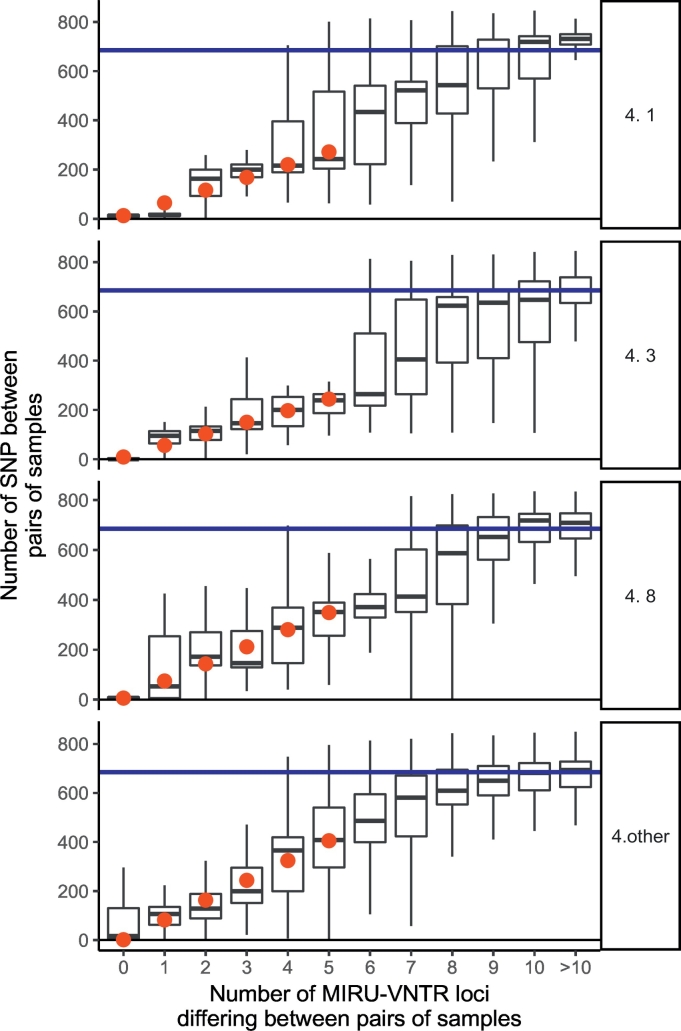
The relationship between MIRU-VNTR profile variation and SNV variation, within lineage 4. The relationship between MIRU-VNTR profile variation and SNV variation, stratified by sublineages of lineage 4, which are shown (e.g. 4.1, 4.3, etc). The x-axis shown the number of MIRU-VNTR loci differing between pairs of isolates. For example, if a sample had a MIRU-VNTR profile of 131, and another 111, one locus differs, which counts as a 1 MIRU-VNTR profile repeat number change. The y-axis shows the median number of SNV in each of a large number of pairs examined. The blue line reflects the median pairwise distance within all sampled isolates of each sublineage. Red dots are fitted median values from a multivariable quantile regression model relating SNV (dependent variable) to lineage, MIRU-VNTR profile difference between 0 and 5 loci, inclusive, and their interaction.

Lineage 4 is a large and complex lineage [17] with global distribution, and contains deep ancestral branches [27]. Our data supports a different relationship between MIRU-VNTR and SNV in the most common sublineages present: 4.1 (*n* = 354), 4.3 (*n* = 158), and 4.8 (*n* = 173), and in other sublineages (*n* = 304): while within all of these groups there was an approximately linear increase in median SNV with MIRU-VNTR differences between 0 and 5 MIRU-VNTR loci (Fig. 5), the slopes observed differed significantly by lineages. For sublineage 4.1, median SNV rose 52, standard error of 0.5 for each MIRU-VNTR locus differing. For lineage 4.3, corresponding slopes were 47 ± 1.3, het*. p* < 10^−6^ relative to lineage 4.1; for sublineage 4.8, 69 ± 0.9, het*. p* < 10^−6^ relative to lineage 4.1, and 81 ± 1.0, het*. p* < 10^−6^ for other lineage 4 isolates.

### 3.6 If MIRU-VNTR is Identical, SNV is Larger in Recent Immigrants than in UK Born Subjects

One possible explanation for the higher SNV seen between pairs of samples in lineages 1–3 when MIRU-VNTR profiles are identical (Fig. 4) would be that (i) lineage 1–3 are more likely to have been acquired abroad and (ii) that more diversity exists abroad within a MIRU-VNTR type than within-country. To test this, we examined individuals who were recorded as having arrived in the country in the last 2 years, vs. individuals who do not fall into this category, i.e. those who are either UK Born or had immigrated >2 years ago.

We tested whether recent migration modified the relationship between MIRU-VNTR type and SNV in the 1792 individuals with lineage 1, 2, 3 or 4 isolation for whom we have data of immigration and UK Birth status (Table 4). Among these cases, we modelled SNV as a function of MIRU-VNTR locus mismatch over 0–3 MIRU-VNTR locus differences, just as illustrated Fig. 4, but included interaction terms allowing both the SNV when there is no MIRU-VNTR difference, and the SNV change per MIRU-VNTR locus difference, to alter. These interaction terms allow us to test whether recent immigration may modify the relationship between MIRU-VNTR difference and SNV.

**Table 4:** Association between immigration status and SNV diversity.

Lineage	Not recent immigrant	Recent immigrant	Estimated Median SNV (95% CI) when MIRU-VNTR identical and neither recent immigrants	Additional change in Median SNV (95% CI) when MIRU-VNTR identical and one or both recent immigrants	Change in Median SNV (95% CI) per MIRU-VNTR locus change when neither are recent migrants	Additional Change in median SNV (95% CI) per MIRU-VNTR locus change when one or both are recent immigrants
1	137	20	175 (149, 290)	40 (32, 53)	-20 (−53, 11)	−17 (−35,47)
2	108	17	172 (168, 176)	10 (6, 14)	15 (7.6,21.4)	−7.5 (−10, −4.8)
3	561	84	104 (99, 109)	16 (1.8, 11.2)	72 (70, 73)	−4.7 (−9.2, −0.19)
4	768	97	9 (8,10)	53 (40, 64)	57 (56, 59)	−14.5 (−19, −10)
Others	23	2	ND			

Median pairwise SNV are significantly higher if one or both samples are from a recent immigrant; this is the case in all lineages (Table 4). For example, in lineage 1 isolates, given MIRU-VNTR identity, the median SNV distance between pairs of samples is 40 SNV higher (95% CI 32,53) if one or both of the pair of samples comes from a recent immigrant. This also occurs in lineage 4: if a pair of samples with MIRU-VNTR identity derives from people who are not recent immigrants, median SNV distance between the pairs is 9 (95% CI 8,10) but if one or more is an immigrant, the median distance is 53 (95% 40,64) higher.

## 4 Discussion

In this prospective study of a cosmopolitan population in the English Midlands, we have quantified how well recent transmission, as defined a range of SNV thresholds, is predicted by shared epidemiological risk factors, by MIRU-VNTR typing, or by a combination of both [21]. We have also demonstrated how lineage strongly affects the performance of MIRU-VNTR-based predictions.

Overall, the PPV for recent transmission, as suggested by close genetic relatedness, for any two isolates with an identical MIRU-VNTR type was only 18.6%. Excluding cases resident at the same address, the PPV varied from as low as 14.8% to 48.0% if shared risk factors were present alongside identical MIRU-VNTR profiles (Fig. 2). However, PPVs for shared MIRU-VNTR profiles differed significantly by lineage, with the strongest associations seen in lineage 4 (European-American), which was also the most frequently observed lineage in the Midlands. The number of patient-to-patient links that need to be investigated to find a single case of recent transmission between non-co-resident individuals with shared MIRU-VNTR types is thus between two and seven, depending on the presence of shared social risk factors.

These data demonstrate that the previous routine practice of grouping samples based on MIRU-VNTR identity, or on a combination of MIRU-VNTR identity and shared epidemiological risk factors, generates highly heterogeneous results, and is likely to contribute to the low cost-effectiveness of MIRU-VNTR typing [8]. Importantly, our data also demonstrate how lineage markedly affects the PPV of MIRU-VNTR links, with the best results seen for lineage 4. Our data support previous work, discussed below, indicating lineage is an important determinant of MIRU-VNTR performance when it used for surveillance reasons.

One possible explanation for why SNV distances between paired isolates sharing a MIRU-VNTR profile within lineages 1, 2 and 3 were greater than for lineage 4 is that the Indo-Oceanic, East-Asian (including Beijing) and East-African Indian lineages are more endemic to countries other than the UK, and that patients diagnosed with these tuberculosis lineages in the UK were infected overseas. Were this the case, pairs of closely genomically related strains would be less likely to be found in isolates from individuals in England, relative to those in the regions were endemic transmission was occurring. Our data supports this: lineage 3 isolates were most common in individuals born in India and Pakistan, relative to other individuals (Table 3). Additionally, recent immigration modifies the MIRU-VNTR: SNP relationship, compatible with a wider pool of variation within a given MIRU-VNTR type in individuals infected abroad, relative to those infected in the UK (Table 4).

A second possible explanation is that the rate of diversification of MIRU-VNTR types relative to SNVs differs between major lineages. Thirdly, MIRU-VNTR variation can result in the same profile via different evolutionary routes (homoplasy) [30], a phenomenon which could also explain the rather flat relationship observed between MIRU-VNTR distance and SNV distance seen in lineages 2 and 3. At least for lineage 2 (Beijing), such homoplasy complicates the ability of MIRU-VNTR to resolve the lineage 2 phylogeny [16, 31]. Whatever the relative importances of these possibilities, our data implies that TB lineages, and their epidemiology, may explain the wide variation in the proportion of TB cases clustering using MIRU-VNTR profiling reported in different settings [10, 32], and the lower coherence of epidemiological risk factors between cases with identical MIRU-VNTR profiles of *M. tuberculosis* isolates from immigrants [15] and those with non-lineage 4, relative to lineage 4 [33].

It was surprising to us that among individuals resident at the same address, only 42% of these pairs were closely genomically linked. One explanation for this relatively low proportion is that some patients from highly endemic countries are likely to co-habit with others from highly endemic countries, potentially increasing the chances of non-clustered isolates, originating from separate exposures, being linked to the same address. Another scenario that could lead to a similar effect would be UK born patients with multiple social risk factors sharing hostels. A third explanation is that even in low incidence countries, the contribution of domestic transmission may have been overestimated historically [33, 34], and may be limited in both low and highly incidence areas [35].

This study relies on data from an accredited clinical MIRU-VNTR typing service. The MIRU-VNTR typing process is complex, and inter-laboratory variation in assay performance has been reported [36]. However, we believe the performance of the MIRU-VNTR typing service described in this paper is similar to that of other clinical services: in the laboratory whose data is reported, the assay had complete concordance with PCR fragment gel sizing both in the published validation assay study [23], and performance in a verification study performed during the described work was very similar to that at validation (Supplementary Data 1). The process was also subject to continuous internal quality control and external quality assessment. These observations support the generalisability of the findings of this work.

An additional limitation, as with other observational epidemiological studies, relates to its uncertain generalisability to other settings with different patterns of transmission, rates of disease, patterns of immigration, and relative prevalence of different lineages. However, the region studied was large and included a mixture of incidence areas, and both urban and rural settings. Another potential limitation is that we cannot be sure that risk factor data was recorded in a fully sensitive manner. Under-ascertainment of risk factor data would reduce the apparent contribution of risk factor data to identifying close genetic neighbours. However, even in the population in which we found in which MIRU-VNTR profiling works best (lineage 4 infections), and in subjects for whom shared risk factors were recorded, the combination of MIRU-VNTR identity and shared risk factors only detects about one in two closely related isolate pairs.

In summary, these data help quantify the limitations of MIRU-VNTR typing for tuberculosis transmission surveillance and control. With routine diagnostic services beginning to transition to WGS technology in multiple high-income countries, as England already has, our data indicates one can expect to see a reduction in the number of potential links requiring epidemiological investigation by a factor of about five. WGS thus stands a much greater chance of contributing to a cost effective control program than MIRU-VNTR typing in low-burden, cosmopolitan settings such as ours, in addition to its value in diagnosis and resistance determination.

## Conflict of Interest Statement

We have no conflicts to declare.

## Author Contributions

Study design: DW, JD, CC; MIRU-VNTR typing: PR, EGS, ER; Performed analyses: JD, DW; Wrote first draft: DW, JD, TW; Critical review of manuscript: all authors.

## Appendix A Supplementary Data

Supplementary material 1 Image 1

Supplementary material 2 Image 2

Supplementary material 3 Image 3
