# Supplementary material for: A Quantitative Evaluation of MIRU-VNTR Typing Against Whole-Genome Sequencing for Identifying Mycobacterium tuberculosis Transmission: A Prospective Observational Cohort Study
Source: eBioMedicine. 2018 Aug 1;34:122–30. doi: 10.1016/j.ebiom.2018.07.019 (PMC6116353; doi:10.1016/j.ebiom.2018.07.019)
Supplement: Supplementary file 1 — Supplementary material 1 [file mmc1.pdf]

Verification of 24 Locus MIRU-VNTR Typing of *Mycobacterium tuberculosis*.

Author: Jason Evans **Date:** 26th November 2014

INTRODUCTION

The PHE Midlands Regional Centre for Mycobacteriology (MRCM) carries out DNA fingerprinting on *M. tuberculosis* strains received from >30 NHS laboratories across the West and East Midlands and South Yorkshire. The MRCM uses a DNA fingerprinting technique called MIRU-VNTR (Mycobacterial Interspersed Repetitive Typing containing Variable Numbers of Tandem Repeats) typing. This method analyses the variation in numbers of DNA repeats sequences in loci located around the *M. tuberculosis* genome. This method initially used five DNA targets, which was subsequently increased to 15 loci in 2003, and the latest generation of this method described here, analyses 24 loci. The incremental increases in the number of loci analysed have increased the discriminatory power of MIRU-VNTR typing and has subsequently increased the concordance between patient social links and molecular data. MIRU-VNTR typing involves: extraction of DNA from a positive culture by sonication and heating; PCR set-up; and VNTR PCR amplicon detection using a Transgenomic WAVE system.

The Transgenomic WAVE system can identify mutations in DNA sequences and can also size DNA sequences. A commercially available size marker (pUC18 plasmid digested with *Hae*III) is available from Transgenomic that can be compared to expected performance data. However, this marker is not suitable for MIRU-VNTR typing as it does not contain enough standards to adequately cover MIRU-VNTR sequences that are a minimum of 51 bp in size. Previous developmental work carried out in the Birmingham laboratory identified WAVE system parameters that provided an accurate and rapid analysis of MIRU-VNTR loci using a molecular standard with more appropriate size intervals (50 bp) (Evans, 2003).

MIRU-VNTR typing using 24 loci was introduced into service by the Birmingham laboratory in 2010. The verification data presented here is a summary of a comparison of data obtained from an EQA panel distributed by the Dutch National TB Reference Laboratory (RIVM) in 2010. The RIVM panel was distributed to ~40 laboratories world-wide as part of an EU-funded project. The RIVM laboratory in the Netherlands is one of the world's leading TB reference laboratories. The RIVM has been analyzing *M. tuberculosis* strains by DNA fingerprinting since 1993. Therefore, analysis of this EQA panel would confirm if the Birmingham laboratory can produce accurate and internationally comparable MIRU-VNTR results.

Prior to this EQA panel, the Birmingham laboratory had evaluated the public health impact of increasing the number of loci from 15 to 24 and found that the increase in loci was justified as it improved the concordance between patient links and molecular data (Evans, 2009).

It was highlighted by CPA in July 2014 that we need an up to date summary of the verification data for 24 locus MIRU-VNTR typing. It was decided in a post CPA meeting in August 2014 that an updated summary would be provided to address this requirement.

The aim of this verification study using an EQA panel was to confirm that the Birmingham laboratory would be able to provide nationally and internationally comparable results.

MATERIALS AND METHODS

The RIVM panel of 30 DNA extracts was received as lyophilised samples which were resuspended in molecular grade water before use. Within the 30 samples, there were 10 pairs of strains with duplicate MIRU-VNTR profiles to assess intra-laboratory reproducibility.

PCR and detection was carried using the following SOPs:
- MI.S1045 - BHH DNA extraction for genotyping, VNTR and MIRU.
- MI.S1057 - Manual BHH 24 Loci MIRU-VNTR PCR set-up.
- MI.S1035 - BHH Wave set up for 9 and 24 loci.

The results obtained by the Midlands Regional Centre for Mycobacteriology (MRCM) were compared to the results provided by the RIVM. Results were obtained by the MRCM in a blinded manner and then submitted to the RIVM. The results were unblinded by the RIVM and compared by the RIVM and a comparison analysis of the results was received back.

Global lineages were assigned using the MIRU-VNTR*plus* website (http://www.miru-vntrplus.org).

An example complete MIRU-VNTR profile is: 32323 2531315311 232243373. Each digit represents a single MIRU-VNTR locus. The complete profiles were compared alongside a comparison of individual loci.

RESULTS

The 30 samples were divided into six batches and at least one of each batch was analysed on each of the three WAVE systems in the Birmingham laboratory between 21st May 2010 and 2nd June 2010 (Table 1).

Table 1. Batch distribution across the three WAVE systems in the MRCM.

Batch	Samples	WAVE	Date of run
1	1-6	2	21/05/2010
2	7-12	3	21/05/2010
3	13-18	3	26/05/2010
4	19-24	3	02/06/2010
5	25-30	1	02/06/2010

Each of the 30 samples had 24 PCR reactions amplified and analysed with a total of 720 PCR reactions amplified and analysed. The Birmingham laboratory successfully amplified 715/720 (99.3%) of all PCRs. However, the concordance between results from Birmingham and the RIVM was only 655/715 (91.6%). There were two MIRU-VNTR loci (VNTR1955 and VNTR4156) where there was a systematic discordance between the two sets of results that meant that each data set was either one repeat higher or one repeat lower than the other data set. Outside of this systematic discordance, there were only two other discordances (Table 2). Please see Appendix I and II for the complete original data for each strain and MIRU-VNTR locus.

The two loci with systematic loci (VNTR1955 and VNTR4156) are part of the additional nine loci that were added onto the existing 15 loci. There were no systematic discordances identified in the 15 loci already routine used.

Table 2. Summary of RIVM EQA panel results.

Description	n (%)
Total number of strains in RIVM EQA panel	30
Total number of PCRs (24 per strain)	720
No. PCRs successfully amplified in Birmingham	715/720 (99.3%)
No. of concordant VNTR loci between MRCM and RIVM	655/715 (91.6%)
No. of concordant VNTR loci after systematic correction	713/715 (99.7%)

The ten paired samples selected to analyse intra-laboratory reproducibility are shown within Table 3. The Birmingham laboratory obtained concordant results within 9/10 (90%) paired samples or 239/240 (99.6%) paired PCR products. The single discordance was between samples 7 and 22 at VNTR1955. Please see Appendix III for the complete original data for each strain.

Table 3. Intra-laboratory reproducibility results.

Pair	DNA sample	Concordant?
6	2	Concordant
7	4	Concordant
2	5	Concordant
10	6	Concordant
8	7	Discordant @ V1955
9	11	Concordant
3	13	Concordant
5	14	Concordant
4	17	Concordant
1	18	Concordant
9	21	Concordant
8	22	Discordant @ V1955
4	23	Concordant
6	24	Concordant
3	25	Concordant
7	26	Concordant
10	27	Concordant
5	28	Concordant
1	29	Concordant
2	30	Concordant

The strain collection in this EQA panel was diverse as all five human global lineages of *M. tuberculosis* were represented alongside two animal lineages of the *M. tuberculosis* complex (Table 4). The most prevalent global clade was the Euro-American lineage.

Table 4. Strain lineage distribution of the EQA panel.

Global Lineage	n (%)
Animal	4 (13)
East African Indian	2 (7)
East Asian	4 (13)
Euro-American	11 (37)
Indo-Oceanic	4 (13)
M. bovis	3 (10)
West African	2 (7)
Total	30 (100)

Discordant Results

There were two MIRU-VNTR loci with systematic discordances for all 30 strains (Appendix I). Birmingham results were systematically one allele lower than the RIVM for locus V1955. Birmingham results were systematically one allele higher than the RIVM for locus V4156.

This systematic discordance was caused by partial repeats as there are partial repeats at the 3' end of most of the 24 MIRU-VNTR loci. The RIVM has an international consensus for MIRU-VNTR analysis where most partial repeats are rounded up or down in a logical manner. However, loci 1955 and 4156 have partial repeats that are not rounded logically (<0.5 repeats rounded up to 1.0 and >0.5 repeats rounded down to 0.0). Therefore, this caused a systematic discordance of either one repeat higher or lower at two loci.

To correct this discordance, the calculation used to calculate the number of repeats was amended so that it conformed to the RIVM international consensus and the EQA panel results were re-analysed and compared.

After correcting this systematic discordance, the Birmingham laboratory obtained concordant results in 713/715 (99.7%) PCR targets.

Remaining Discordant Results

After the systematic correction, there were two remaining discordant results. ETR-B in sample 12 where Birmingham called six repeats and the RIVM called four repeats and VNTR1955 in sample 22 where Birmingham called two repeats and the RIVM called 14 repeats.

In sample 12, six repeats were obtained again on repeat analysis. Comparison of our results with another UK laboratory identified that the other UK laboratory obtained two repeats at V1955 for sample 12 which indicates that this may have been a problematic locus to identify.

In sample 22, the peak at two repeats was very low in intensity (<1mV) and should not have been called a peak. Peaks have a cut-off threshold of 1 mV and this peak was below this.

Intra-laboratory reproducibility

Within the panel of 30 DNA extracts, there were 10 pairs of duplicate extracts to test intra-laboratory reproducibility. The 10 pairs equated to 480 individual PCRs. The Birmingham laboratory successfully amplified a product in 479/480 (99.8%) PCRs. The Birmingham laboratory obtained concordant results for 478/479 (99.8%) PCRs or 9/10 paired samples. The sole discordance was VNTR1955 in samples 7 and 22. The correct result was 14 repeats but Birmingham failed to amplify sample 7 and obtained a peak

and one repeat in sample 22. Upon further review, the peak at one repeat in sample 22 was very low in intensity and probably should not have been called a peak.

DISCUSSION

Within our verification study, we identified a systematic variation between the Birmingham and RIVM methods of calling MIRU-VNTR alleles at two loci. Upon correction and review of discordant results, only one discordant result could not be resolved (Locus V1955 in sample 12).

The two loci with systematic loci (VNTR1955 and VNTR4156) are part of the additional nine loci that were added onto the existing 15 loci to increase the discriminatory power of the typing method. Routine reporting of the additional nine loci including VNTR1955 and VNTR4156 commenced only after this systematic discordance had been identified and corrected. Previous EQA panels using 15 loci showed that we were reporting nationally and internationally comparable MIRU-VNTR results for 15 loci.

Previous studies have shown that increasing the number of loci analysed from 15 to 24 increases the discriminatory power of MIRU-VNTR typing and increases the numbers of patients correctly clustered by molecular methods (Oelemann, 2007; Supply, 2006). We analysed seven clusters that contained 71 strains and found that increasing the number of loci from 15 to 24 increased the concordance between patient social links and molecular data from 44/71(62%) to 66/71(93%) patients (Evans, 2009).

The data presented here shows that the Birmingham laboratory successfully amplified a high proportion of MIRU-VNTR loci (>99%) and achieved a high degree of concordance (>99%) with an international set of strains from another laboratory.

Previous developmental work carried out in the Birmingham laboratory identified WAVE system parameters that generated accurate and rapid analysis of MIRU-VNTR loci. The additional nine loci evaluated here have similar DNA sequences when compared to the existing 15 loci. Therefore, no amendment in WAVE system parameters was required to provide highly comparable results in this EQA panel.

The strain collection in this EQA panel was diverse as all of the five human global lineages of *M. tuberculosis* were included alongside two animal lineages. The predominant lineage was the Euro-American lineage. This closely reflects the strain distribution observed in the Midlands (Evans, 2010).

CONCLUSION

After correction of a systematic discordance which affected two MIRU-VNTR loci, we verified that the Birmingham MIRU-VNTR assay can accurately identify MIRU-VNTR allele copy number in DNA extracted from *M. tuberculosis* complex strains.

This level of performance identified in this dataset is acceptable.

LIMITATIONS

Unlike NEQAS, the comparison within this EQA panel was solely against results from one laboratory. NEQAS panels analyse participant results and exclude samples from scoring that have been difficult to analyse for participating laboratories where a consensus has been difficult to achieve. In the RIVM panel, one of the discordant loci was apparently problematic for another UK laboratory as well.

The strain collection in this EQA panel was diverse with wide allele ranges (0-14 repeats). However, it has not been ascertained if any of these strains have ever been identified in the UK.

We do not routinely quantify the DNA concentration in our extracts. The high PCR amplification rate in this EQA panel (>99%) indicates that the DNA quality and quantity may have been better and higher respectively than is usually seen from routine positive cultures.

REFERENCES

Evans JT, Hawkey PM, Smith EG, Boese KA, Warren RE, Hong G. Automated high-throughput mycobacterial interspersed repetitive unit typing of Mycobacterium tuberculosis strains by a combination of PCR and nondenaturing high-performance liquid chromatography. *J Clin Microbiol.* 2004 **42(9)**:4175-80.

Evans JT, Taylor B, Estephane D, Gardiner S, Smith EG, Hawkey PM. Application of the optimal 24 MIRU-VNTR loci set for *Mycobacterium tuberculosis* strains improves the correlation between strain typing and epidemiological data. *Clin Microbiol Infect.* 2009 **15(S4)**:134.

Evans JT, Gardiner S, Smith EG, Webber R, Hawkey PM. Global Origin of *Mycobacterium tuberculosis* in the Midlands, UK. *Emerg Infect Dis.* 2010 16(3):542-5.

Oelemann MC, Diel R, Vatin V, Haas W, Rüsch-Gerdes S, Locht C, Niemann S, Supply P. Assessment of an optimized mycobacterial interspersed repetitive-unit-variable-number tandem-repeat typing system combined with spoligotyping for population-based molecular epidemiology studies of tuberculosis. *J Clin Microbiol.* 2007 45(3):691-7.

Supply P, Allix C, Lesjean S, Cardoso-Oelemann M, Rüsch-Gerdes S, Willery E, Savine E, de Haas P, van Deutekom H, Roring S, Bifani P, Kurepina N, Kreiswirth B, Sola C, Rastogi N, Vatin V, Gutierrez MC, Fauville M, Niemann S, Skuce R, Kremer K, Locht C, van Soolingen D. Proposal for standardization of optimized mycobacterial interspersed repetitive unit-variable-number tandem repeat typing of *Mycobacterium tuberculosis*. *J Clin Microbiol.* 2006 44(12):4498-510.

APPENDIX

Appendix I. Comparison of RIVM and Birmingham results for all 30 samples before systematic correction. Differences between RIVM and Birmingham results are highlighted in grey. "-" indicate a failed PCR. Failed PCRs were not considered as discordances.

#	Lab	ETR-A	ETR-B	ETR-C	ETR-D	ETR-E	M02	M10	M16	M20	M23	M24	M26	M27	M39	M40	V0424	V1955	V2163b	V2347	V2401	V3171	V3690	V4052	V4156
15	Loci	Y	Y	Y	Y	Y	Y	Y	Y	Y	Y	Y	Y	Y	Y	Y	N	N	N	N	N	N	N	N	N
24	Loci	Y	Y	Y	Y	Y	Y	Y	Y	Y	Y	Y	Y	Y	Y	Y	Y	Y	Y	Y	Y	Y	Y	Y	Y
1	BIR	3	2	3	3	3	2	5	3	1	3	1	5	3	1	1	2	2	2	2	4	3	3	7	4
1	RIVM	3	2	3	3	3	2	5	3	1	3	1	5	3	1	1	2	3	2	2	4	3	3	7	3
1	Match	Y	Y	Y	Y	Y	Y	Y	Y	Y	Y	Y	Y	Y	Y	Y	Y	N	Y	Y	Y	Y	Y	Y	N
2	BIR	4	2	4	3	6	2	3	3	2	5	1	7	3	3	3	4	4	6	4	4	3	3	7	3
2	RIVM	4	2	4	3	6	2	3	3	2	5	1	7	3	3	3	4	5	6	4	4	3	3	7	2
2	Match	Y	Y	Y	Y	Y	Y	Y	Y	Y	Y	Y	Y	Y	Y	Y	Y	N	Y	Y	Y	Y	Y	Y	N
3	BIR	4	2	4	3	5	2	3	3	2	5	1	8	3	3	3	3	4	4	4	4	4	3	8	3
3	RIVM	4	2	4	3	5	2	3	3	2	5	1	8	3	3	3	3	5	4	4	4	4	3	8	2
3	Match	Y	Y	Y	Y	Y	Y	Y	Y	Y	Y	Y	Y	Y	Y	Y	Y	N	Y	Y	Y	Y	Y	Y	N
4	BIR	3	3	4	4'	3	2	3	2	2	6	1	3	3	2	1	2	1	5	4	2	3	5	5	3
4	RIVM	3	3	4	4'	3	2	3	2	2	6	1	3	3	2	1	2	2	5	4	2	3	5	5	2
4	Match	Y	Y	Y	Y	Y	Y	Y	Y	Y	Y	Y	Y	Y	Y	Y	Y	N	Y	Y	Y	Y	Y	Y	N
5	BIR	5	5	6	3'	3	2	2	3	2	4	2	5	3	2	2	0	0	3	2	2	3	2	5	1
5	RIVM	5	5	6	3'	3	2	2	3	2	4	2	5	3	2	2	0	1	3	2	2	3	2	5	0
5	Match	Y	Y	Y	Y	Y	Y	Y	Y	Y	Y	Y	Y	Y	Y	Y	Y	N	Y	Y	Y	Y	Y	Y	N
6	BIR	8	3	5	7	1	2	7	3	1	4	2	2	2	2	2	3	2	7	3	2	4	3	7	4
6	RIVM	8	3	5	7	1	2	7	3	1	4	2	2	2	2	2	3	3	7	3	2	4	3	7	3
6	Match	Y	Y	Y	Y	Y	Y	Y	Y	Y	Y	Y	Y	Y	Y	Y	Y	N	Y	Y	Y	Y	Y	Y	N
7	BIR	6	4	4	6	6	2	4	3	2	10	2	2	3	3	3	2	-	3	3	1	3	6	6	2
7	RIVM	6	4	4	6	6	2	4	3	2	10	2	2	3	3	3	2	14	3	3	1	3	6	6	1
7	Match	Y	Y	Y	Y	Y	Y	Y	Y	Y	Y	Y	Y	Y	Y	Y	Y	-	Y	Y	Y	Y	Y	Y	N

#	Lab	ETR-A	ETR-B	ETR-C	ETR-D	ETR-E	M02	M10	M16	M20	M23	M24	M26	M27	M39	M40	V0424	V1955	V2163b	V2347	V2401	V3171	V3690	V4052	V4156
8	BIR	4	2	3	3	5	2	3	3	2	5	1	4	3	3	3	3	1	3	4	2	3	3	6	3
8	RIVM	4	2	3	3	5	2	3	3	2	5	1	4	3	3	3	3	2	3	4	2	3	3	6	2
8	Match	Y	Y	Y	Y	Y	Y	Y	Y	Y	Y	Y	Y	Y	Y	Y	Y	N	Y	Y	Y	Y	Y	Y	N
9	BIR	2	2	4	3	3	2	3	3	2	6	1	3	3	2	1	4	2	2	4	2	1	1	6	3
9	RIVM	2	2	4	3	3	2	3	3	2	6	1	3	3	2	1	4	3	2	4	2	1	1	6	2
9	Match	Y	Y	Y	Y	Y	Y	Y	Y	Y	Y	Y	Y	Y	Y	Y	Y	N	Y	Y	Y	Y	Y	Y	N
10	BIR	3	2	3	3	3	2	5	3	2	5	1	5	3	2	3	2	2	5	4	4	3	3	4	4
10	RIVM	3	2	3	3	3	2	5	3	2	5	1	5	3	2	3	2	3	5	4	4	3	3	4	3
10	Match	Y	Y	Y	Y	Y	Y	Y	Y	Y	Y	Y	Y	Y	Y	Y	Y	N	Y	Y	Y	Y	Y	Y	N
11	BIR	5	4	4	6	6	2	4	3	2	11	2	2	3	3	3	3	7	3	3	1	3	4	6	2
11	RIVM	5	4	4	6	6	2	4	3	2	11	2	2	3	3	3	3	8	3	3	1	3	4	6	1
11	Match	Y	Y	Y	Y	Y	Y	Y	Y	Y	Y	Y	Y	Y	Y	Y	Y	N	Y	Y	Y	Y	Y	Y	N
12	BIR	9	6	4	6	3	2	6	4	2	4	2	2	2	2	1	3	3	9	3	4	3	3	7	1
12	RIVM	9	4	4	6	3	2	6	4	2	4	2	2	2	2	1	3	4	9	3	4	3	3	7	0
12	Match	Y	N	Y	Y	Y	Y	Y	Y	Y	Y	Y	Y	Y	Y	Y	Y	N	Y	Y	Y	Y	Y	Y	N
13	BIR	3	1	4	3	2	2	2	3	2	5	1	5	3	2	3	2	2	4	4	2	3	3	7	3
13	RIVM	3	1	4	3	2	2	2	3	2	5	1	5	3	2	3	2	3	4	4	2	3	3	7	2
13	Match	Y	Y	Y	Y	Y	Y	Y	Y	Y	Y	Y	Y	Y	Y	Y	Y	N	Y	Y	Y	Y	Y	Y	N
14	BIR	3	2	2	3	5	2	6	4	2	5	1	7	3	3	2	8	3	2	4	2	3	3	6	5
14	RIVM	3	2	2	3	5	2	6	4	2	5	1	7	3	3	2	8	4	2	4	2	3	3	6	4
14	Match	Y	Y	Y	Y	Y	Y	Y	Y	Y	Y	Y	Y	Y	Y	Y	Y	N	Y	Y	Y	Y	Y	Y	N
15	BIR	4	2	4	3	5	2	3	3	1	5	1	5	3	4	4	4	4	5	4	4	3	3	8	3
15	RIVM	4	2	4	3	5	2	3	3	1	5	1	5	3	4	4	4	5	5	4	4	3	3	8	2
15	Match	Y	Y	Y	Y	Y	Y	Y	Y	Y	Y	Y	Y	Y	Y	Y	Y	N	Y	Y	Y	Y	Y	Y	N
16	BIR	4	2	5	3	-	2	3	3	2	5	1	5	3	2	2	1	1	3	4	2	3	3	7	3
16	RIVM	4	2	5	3	5	2	3	3	2	5	1	5	3	2	2	1	2	3	4	2	3	3	7	2
16	Match	Y	Y	Y	Y	-	Y	Y	Y	Y	Y	Y	Y	Y	Y	Y	Y	N	Y	Y	Y	Y	Y	Y	N
17	BIR	2	2	2	3	3	2	4	2	2	6	1	6	3	2	1	4	3	4	4	1	3	2	8	3
17	RIVM	2	2	2	3	3	2	4	2	2	6	1	6	3	2	1	4	4	4	4	1	3	2	8	2

#	Lab	ETR-A	ETR-B	ETR-C	ETR-D	ETR-E	M02	M10	M16	M20	M23	M24	M26	M27	M39	M40	V0424	V1955	V2163b	V2347	V2401	V3171	V3690	V4052	V4156
17	Match	Y	Y	Y	Y	Y	Y	Y	Y	Y	Y	Y	Y	Y	Y	Y	Y	N	Y	Y	Y	Y	Y	Y	N
18	BIR	6	4	5	2	5	2	5	4	2	4	2	5	3	2	2	3	3	5	3	4	3	4	6	4
18	RIVM	6	4	5	2	5	2	5	4	2	4	2	5	3	2	2	3	4	5	3	4	3	4	6	3
18	Match	Y	Y	Y	Y	Y	Y	Y	Y	Y	Y	Y	Y	Y	Y	Y	Y	N	Y	Y	Y	Y	Y	Y	N
19	BIR	-	4	6	3	4	3	3	2	1	2	6	3	2	2	8	3	2	-	5	2	4	2	-	2
19	RIVM	10	4	6	3	4	3	3	2	1	2	6	3	2	2	8	3	3	11	5	2	4	2	4	1
19	Match	-	Y	Y	Y	Y	Y	Y	Y	Y	Y	Y	Y	Y	Y	Y	Y	N	-	Y	Y	Y	Y	-	N
20	BIR	5	1	5	4	3	2	2	3	2	4	2	5	3	2	2	1	1	2	3	4	3	2	7	2
20	RIVM	5	1	5	4	3	2	2	3	2	4	2	5	3	2	2	1	2	2	3	4	3	2	7	1
20	Match	Y	Y	Y	Y	Y	Y	Y	Y	Y	Y	Y	Y	Y	Y	Y	Y	N	Y	Y	Y	Y	Y	Y	N
21	BIR	5	4	4	6	6	2	4	3	2	11	2	2	3	3	3	3	7	3	3	1	3	4	6	2
21	RIVM	5	4	4	6	6	2	4	3	2	11	2	2	3	3	3	3	8	3	3	1	3	4	6	1
21	Match	Y	Y	Y	Y	Y	Y	Y	Y	Y	Y	Y	Y	Y	Y	Y	Y	N	Y	Y	Y	Y	Y	Y	N
22	BIR	6	4	4	6	6	2	4	3	2	10	2	2	3	3	3	2	1	3	3	1	3	6	6	2
22	RIVM	6	4	4	6	6	2	4	3	2	10	2	2	3	3	3	2	14	3	3	1	3	6	6	1
22	Match	Y	Y	Y	Y	Y	Y	Y	Y	Y	Y	Y	Y	Y	Y	Y	Y	N	Y	Y	Y	Y	Y	Y	N
23	BIR	2	2	2	3	3	2	4	2	2	6	1	6	3	2	1	4	3	4	4	1	3	2	8	3
23	RIVM	2	2	2	3	3	2	4	2	2	6	1	6	3	2	1	4	4	4	4	1	3	2	8	2
23	Match	Y	Y	Y	Y	Y	Y	Y	Y	Y	Y	Y	Y	Y	Y	Y	Y	N	Y	Y	Y	Y	Y	Y	N
24	BIR	4	2	4	3	6	2	3	3	2	5	1	7	3	3	3	4	4	6	4	4	3	3	7	3
24	RIVM	4	2	4	3	6	2	3	3	2	5	1	7	3	3	3	4	5	6	4	4	3	3	7	2
24	Match	Y	Y	Y	Y	Y	Y	Y	Y	Y	Y	Y	Y	Y	Y	Y	Y	N	Y	Y	Y	Y	Y	Y	N
25	BIR	3	1	4	3	2	2	2	3	2	5	1	5	3	2	3	2	2	4	4	2	3	3	7	3
25	RIVM	3	1	4	3	2	2	2	3	2	5	1	5	3	2	3	2	3	4	4	2	3	3	7	2
25	Match	Y	Y	Y	Y	Y	Y	Y	Y	Y	Y	Y	Y	Y	Y	Y	Y	N	Y	Y	Y	Y	Y	Y	N
26	BIR	3	3	4	4’	3	2	3	2	2	6	1	3	3	2	1	2	1	5	4	2	3	5	5	3
26	RIVM	3	3	4	4’	3	2	3	2	2	6	1	3	3	2	1	2	2	5	4	2	3	5	5	2
26	Match	Y	Y	Y	Y	Y	Y	Y	Y	Y	Y	Y	Y	Y	Y	Y	Y	N	Y	Y	Y	Y	Y	Y	N
27	BIR	8	3	5	7	1	2	7	3	1	4	2	2	2	2	2	3	2	7	3	2	4	3	7	4

#	Lab	ETR-A	ETR-B	ETR-C	ETR-D	ETR-E	M02	M10	M16	M20	M23	M24	M26	M27	M39	M40	V0424	V1955	V2163b	V2347	V2401	V3171	V3690	V4052	V4156
27	RIVM	8	3	5	7	1	2	7	3	1	4	2	2	2	2	2	3	3	7	3	2	4	3	7	3
27	Match	Y	Y	Y	Y	Y	Y	Y	Y	Y	Y	Y	Y	Y	Y	Y	Y	N	Y	Y	Y	Y	Y	Y	N
28	BIR	3	2	2	3	5	2	6	4	2	5	1	7	3	3	2	8	3	2	4	2	3	3	6	5
28	RIVM	3	2	2	3	5	2	6	4	2	5	1	7	3	3	2	8	4	2	4	2	3	3	6	4
28	Match	Y	Y	Y	Y	Y	Y	Y	Y	Y	Y	Y	Y	Y	Y	Y	Y	N	Y	Y	Y	Y	Y	Y	N
29	BIR	6	4	5	2	5	2	5	4	2	4	2	5	3	2	2	3	3	5	3	4	3	4	6	4
29	RIVM	6	4	5	2	5	2	5	4	2	4	2	5	3	2	2	3	4	5	3	4	3	4	6	3
29	Match	Y	Y	Y	Y	Y	Y	Y	Y	Y	Y	Y	Y	Y	Y	Y	Y	N	Y	Y	Y	Y	Y	Y	N
30	BIR	5	5	6	3’	3	2	2	3	2	4	2	5	3	2	2	0	0	3	2	2	3	2	5	1
30	RIVM	5	5	6	3’	3	2	2	3	2	4	2	5	3	2	2	0	1	3	2	2	3	2	5	0
30	Match	Y	Y	Y	Y	Y	Y	Y	Y	Y	Y	Y	Y	Y	Y	Y	Y	N	Y	Y	Y	Y	Y	Y	N

Appendix II. Comparison of RIVM and Birmingham results for all 30 samples after systematic correction. Locus V1955 was increased by one allele and locus V4156 was reduced by one allele. Differences between RIVM and Birmingham results are highlighted in grey. “-“ indicate a failed PCR. Failed PCRs were not considered as discordances.

#	Lab	ETR-A	ETR-B	ETR-C	ETR-D	ETR-E	M02	M10	M16	M20	M23	M24	M26	M27	M39	M40	V0424	V1955	V2163b	V2347	V2401	V3171	V3690	V4052	V4156
15	Loci	Y	Y	Y	Y	Y	Y	Y	Y	Y	Y	Y	Y	Y	Y	Y	N	N	N	N	N	N	N	N	N
24	Loci	Y	Y	Y	Y	Y	Y	Y	Y	Y	Y	Y	Y	Y	Y	Y	Y	Y	Y	Y	Y	Y	Y	Y	Y
1	BIR	3	2	3	3	3	2	5	3	1	3	1	5	3	1	1	2	3	2	2	4	3	3	7	3
1	RIVM	3	2	3	3	3	2	5	3	1	3	1	5	3	1	1	2	3	2	2	4	3	3	7	3
1	Match	Y	Y	Y	Y	Y	Y	Y	Y	Y	Y	Y	Y	Y	Y	Y	Y	Y	Y	Y	Y	Y	Y	Y	Y
2	BIR	4	2	4	3	6	2	3	3	2	5	1	7	3	3	3	4	5	6	4	4	3	3	7	2
2	RIVM	4	2	4	3	6	2	3	3	2	5	1	7	3	3	3	4	5	6	4	4	3	3	7	2
2	Match	Y	Y	Y	Y	Y	Y	Y	Y	Y	Y	Y	Y	Y	Y	Y	Y	Y	Y	Y	Y	Y	Y	Y	Y
3	BIR	4	2	4	3	5	2	3	3	2	5	1	8	3	3	3	3	5	4	4	4	4	3	8	2
3	RIVM	4	2	4	3	5	2	3	3	2	5	1	8	3	3	3	3	5	4	4	4	4	3	8	2
3	Match	Y	Y	Y	Y	Y	Y	Y	Y	Y	Y	Y	Y	Y	Y	Y	Y	Y	Y	Y	Y	Y	Y	Y	Y
4	BIR	3	3	4	4’	3	2	3	2	2	6	1	3	3	2	1	2	2	5	4	2	3	5	5	2
4	RIVM	3	3	4	4’	3	2	3	2	2	6	1	3	3	2	1	2	2	5	4	2	3	5	5	2
4	Match	Y	Y	Y	Y	Y	Y	Y	Y	Y	Y	Y	Y	Y	Y	Y	Y	Y	Y	Y	Y	Y	Y	Y	Y
5	BIR	5	5	6	3’	3	2	2	3	2	4	2	5	3	2	2	0	1	3	2	2	3	2	5	0
5	RIVM	5	5	6	3’	3	2	2	3	2	4	2	5	3	2	2	0	1	3	2	2	3	2	5	0
5	Match	Y	Y	Y	Y	Y	Y	Y	Y	Y	Y	Y	Y	Y	Y	Y	Y	Y	Y	Y	Y	Y	Y	Y	Y
6	BIR	8	3	5	7	1	2	7	3	1	4	2	2	2	2	2	3	3	7	3	2	4	3	7	3
6	RIVM	8	3	5	7	1	2	7	3	1	4	2	2	2	2	2	3	3	7	3	2	4	3	7	3
6	Match	Y	Y	Y	Y	Y	Y	Y	Y	Y	Y	Y	Y	Y	Y	Y	Y	Y	Y	Y	Y	Y	Y	Y	Y
7	BIR	6	4	4	6	6	2	4	3	2	10	2	2	3	3	3	2	-	3	3	1	3	6	6	1
7	RIVM	6	4	4	6	6	2	4	3	2	10	2	2	3	3	3	2	14	3	3	1	3	6	6	1
7	Match	Y	Y	Y	Y	Y	Y	Y	Y	Y	Y	Y	Y	Y	Y	Y	Y	-	Y	Y	Y	Y	Y	Y	Y
8	BIR	4	2	3	3	5	2	3	3	2	5	1	4	3	3	3	3	2	3	4	2	3	3	6	2
8	RIVM	4	2	3	3	5	2	3	3	2	5	1	4	3	3	3	3	2	3	4	2	3	3	6	2

#	Lab	ETR-A	ETR-B	ETR-C	ETR-D	ETR-E	M02	M10	M16	M20	M23	M24	M26	M27	M39	M40	V0424	V1955	V2163b	V2347	V2401	V3171	V3690	V4052	V4156
8	Match	Y	Y	Y	Y	Y	Y	Y	Y	Y	Y	Y	Y	Y	Y	Y	Y	Y	Y	Y	Y	Y	Y	Y	Y
9	BIR	2	2	4	3	3	2	3	3	2	6	1	3	3	2	1	4	3	2	4	2	1	1	6	2
9	RIVM	2	2	4	3	3	2	3	3	2	6	1	3	3	2	1	4	3	2	4	2	1	1	6	2
9	Match	Y	Y	Y	Y	Y	Y	Y	Y	Y	Y	Y	Y	Y	Y	Y	Y	Y	Y	Y	Y	Y	Y	Y	Y
10	BIR	3	2	3	3	3	2	5	3	2	5	1	5	3	2	3	2	3	5	4	4	3	3	4	3
10	RIVM	3	2	3	3	3	2	5	3	2	5	1	5	3	2	3	2	3	5	4	4	3	3	4	3
10	Match	Y	Y	Y	Y	Y	Y	Y	Y	Y	Y	Y	Y	Y	Y	Y	Y	Y	Y	Y	Y	Y	Y	Y	Y
11	BIR	5	4	4	6	6	2	4	3	2	11	2	2	3	3	3	3	8	3	3	1	3	4	6	2
11	RIVM	5	4	4	6	6	2	4	3	2	11	2	2	3	3	3	3	8	3	3	1	3	4	6	2
11	Match	Y	Y	Y	Y	Y	Y	Y	Y	Y	Y	Y	Y	Y	Y	Y	Y	Y	Y	Y	Y	Y	Y	Y	Y
12	BIR	9	6	4	6	3	2	6	4	2	4	2	2	2	2	1	3	4	9	3	4	3	3	7	0
12	RIVM	9	4	4	6	3	2	6	4	2	4	2	2	2	2	1	3	4	9	3	4	3	3	7	0
12	Match	Y	N	Y	Y	Y	Y	Y	Y	Y	Y	Y	Y	Y	Y	Y	Y	Y	Y	Y	Y	Y	Y	Y	Y
13	BIR	3	1	4	3	2	2	2	3	2	5	1	5	3	2	3	2	3	4	4	2	3	3	7	2
13	RIVM	3	1	4	3	2	2	2	3	2	5	1	5	3	2	3	2	3	4	4	2	3	3	7	2
13	Match	Y	Y	Y	Y	Y	Y	Y	Y	Y	Y	Y	Y	Y	Y	Y	Y	Y	Y	Y	Y	Y	Y	Y	Y
14	BIR	3	2	2	3	5	2	6	4	2	5	1	7	3	3	2	8	4	2	4	2	3	3	6	4
14	RIVM	3	2	2	3	5	2	6	4	2	5	1	7	3	3	2	8	4	2	4	2	3	3	6	4
14	Match	Y	Y	Y	Y	Y	Y	Y	Y	Y	Y	Y	Y	Y	Y	Y	Y	Y	Y	Y	Y	Y	Y	Y	Y
15	BIR	4	2	4	3	5	2	3	3	1	5	1	5	3	4	4	4	5	5	4	4	3	3	8	2
15	RIVM	4	2	4	3	5	2	3	3	1	5	1	5	3	4	4	4	5	5	4	4	3	3	8	2
15	Match	Y	Y	Y	Y	Y	Y	Y	Y	Y	Y	Y	Y	Y	Y	Y	Y	Y	Y	Y	Y	Y	Y	Y	Y
16	BIR	4	2	5	3	-	2	3	3	2	5	1	5	3	2	2	1	2	3	4	2	3	3	7	2
16	RIVM	4	2	5	3	5	2	3	3	2	5	1	5	3	2	2	1	2	3	4	2	3	3	7	2
16	Match	Y	Y	Y	Y	-	Y	Y	Y	Y	Y	Y	Y	Y	Y	Y	Y	Y	Y	Y	Y	Y	Y	Y	Y
17	BIR	2	2	2	3	3	2	4	2	2	6	1	6	3	2	1	4	4	4	4	1	3	2	8	2
17	RIVM	2	2	2	3	3	2	4	2	2	6	1	6	3	2	1	4	4	4	4	1	3	2	8	2
17	Match	Y	Y	Y	Y	Y	Y	Y	Y	Y	Y	Y	Y	Y	Y	Y	Y	Y	Y	Y	Y	Y	Y	Y	Y
18	BIR	6	4	5	2	5	2	5	4	2	4	2	5	3	2	2	3	4	5	3	4	3	4	6	3

#	Lab	ETR-A	ETR-B	ETR-C	ETR-D	ETR-E	M02	M10	M16	M20	M23	M24	M26	M27	M39	M40	V0424	V1955	V2163b	V2347	V2401	V3171	V3690	V4052	V4156
18	RIVM	6	4	5	2	5	2	5	4	2	4	2	5	3	2	2	3	4	5	3	4	3	4	6	3
18	Match	Y	Y	Y	Y	Y	Y	Y	Y	Y	Y	Y	Y	Y	Y	Y	Y	Y	Y	Y	Y	Y	Y	Y	Y
19	BIR	-	4	6	3	4	3	3	2	1	2	6	3	2	2	8	3	3	-	5	2	4	2	-	1
19	RIVM	10	4	6	3	4	3	3	2	1	2	6	3	2	2	8	3	3	11	5	2	4	2	4	1
19	Match	-	Y	Y	Y	Y	Y	Y	Y	Y	Y	Y	Y	Y	Y	Y	Y	Y	-	Y	Y	Y	Y	-	Y
20	BIR	5	1	5	4	3	2	2	3	2	4	2	5	3	2	2	1	2	2	3	4	3	2	7	1
20	RIVM	5	1	5	4	3	2	2	3	2	4	2	5	3	2	2	1	2	2	3	4	3	2	7	1
20	Match	Y	Y	Y	Y	Y	Y	Y	Y	Y	Y	Y	Y	Y	Y	Y	Y	Y	Y	Y	Y	Y	Y	Y	Y
21	BIR	5	4	4	6	6	2	4	3	2	11	2	2	3	3	3	3	8	3	3	1	3	4	6	1
21	RIVM	5	4	4	6	6	2	4	3	2	11	2	2	3	3	3	3	8	3	3	1	3	4	6	1
21	Match	Y	Y	Y	Y	Y	Y	Y	Y	Y	Y	Y	Y	Y	Y	Y	Y	Y	Y	Y	Y	Y	Y	Y	Y
22	BIR	6	4	4	6	6	2	4	3	2	10	2	2	3	3	3	2	2	3	3	1	3	6	6	1
22	RIVM	6	4	4	6	6	2	4	3	2	10	2	2	3	3	3	2	14	3	3	1	3	6	6	1
22	Match	Y	Y	Y	Y	Y	Y	Y	Y	Y	Y	Y	Y	Y	Y	Y	Y	N	Y	Y	Y	Y	Y	Y	Y
23	BIR	2	2	2	3	3	2	4	2	2	6	1	6	3	2	1	4	4	4	4	1	3	2	8	2
23	RIVM	2	2	2	3	3	2	4	2	2	6	1	6	3	2	1	4	4	4	4	1	3	2	8	2
23	Match	Y	Y	Y	Y	Y	Y	Y	Y	Y	Y	Y	Y	Y	Y	Y	Y	Y	Y	Y	Y	Y	Y	Y	Y
24	BIR	4	2	4	3	6	2	3	3	2	5	1	7	3	3	3	4	5	6	4	4	3	3	7	2
24	RIVM	4	2	4	3	6	2	3	3	2	5	1	7	3	3	3	4	5	6	4	4	3	3	7	2
24	Match	Y	Y	Y	Y	Y	Y	Y	Y	Y	Y	Y	Y	Y	Y	Y	Y	Y	Y	Y	Y	Y	Y	Y	Y
25	BIR	3	1	4	3	2	2	2	3	2	5	1	5	3	2	3	2	3	4	4	2	3	3	7	2
25	RIVM	3	1	4	3	2	2	2	3	2	5	1	5	3	2	3	2	3	4	4	2	3	3	7	2
25	Match	Y	Y	Y	Y	Y	Y	Y	Y	Y	Y	Y	Y	Y	Y	Y	Y	Y	Y	Y	Y	Y	Y	Y	Y
26	BIR	3	3	4	4’	3	2	3	2	2	6	1	3	3	2	1	2	2	5	4	2	3	5	5	2
26	RIVM	3	3	4	4’	3	2	3	2	2	6	1	3	3	2	1	2	2	5	4	2	3	5	5	2
26	Match	Y	Y	Y	Y	Y	Y	Y	Y	Y	Y	Y	Y	Y	Y	Y	Y	Y	Y	Y	Y	Y	Y	Y	Y
27	BIR	8	3	5	7	1	2	7	3	1	4	2	2	2	2	2	3	3	7	3	2	4	3	7	3
27	RIVM	8	3	5	7	1	2	7	3	1	4	2	2	2	2	2	3	3	7	3	2	4	3	7	3
27	Match	Y	Y	Y	Y	Y	Y	Y	Y	Y	Y	Y	Y	Y	Y	Y	Y	Y	Y	Y	Y	Y	Y	Y	Y

#	Lab	ETR-A	ETR-B	ETR-C	ETR-D	ETR-E	M02	M10	M16	M20	M23	M24	M26	M27	M39	M40	V0424	V1955	V2163b	V2347	V2401	V3171	V3690	V4052	V4156
28	BIR	3	2	2	3	5	2	6	4	2	5	1	7	3	3	2	8	4	2	4	2	3	3	6	4
28	RIVM	3	2	2	3	5	2	6	4	2	5	1	7	3	3	2	8	4	2	4	2	3	3	6	4
28	Match	Y	Y	Y	Y	Y	Y	Y	Y	Y	Y	Y	Y	Y	Y	Y	Y	Y	Y	Y	Y	Y	Y	Y	Y
29	BIR	6	4	5	2	5	2	5	4	2	4	2	5	3	2	2	3	4	5	3	4	3	4	6	3
29	RIVM	6	4	5	2	5	2	5	4	2	4	2	5	3	2	2	3	4	5	3	4	3	4	6	3
29	Match	Y	Y	Y	Y	Y	Y	Y	Y	Y	Y	Y	Y	Y	Y	Y	Y	Y	Y	Y	Y	Y	Y	Y	Y
30	BIR	5	5	6	3’	3	2	2	3	2	4	2	5	3	2	2	0	1	3	2	2	3	2	5	0
30	RIVM	5	5	6	3’	3	2	2	3	2	4	2	5	3	2	2	0	1	3	2	2	3	2	5	0
30	Match	Y	Y	Y	Y	Y	Y	Y	Y	Y	Y	Y	Y	Y	Y	Y	Y	Y	Y	Y	Y	Y	Y	Y	Y

Appendix III. Original data for 10 pairs of strains used to assess intra-laboratory reproducibility.

Pair	DNA sample	Lab	ETR	MIRU	VNTR+	Concordant?
1	18	BIR	64525	2542425322	345343463	
1	18	RIVM	64525	2542425322	345343463	
1	18	Matching?	TRUE	TRUE	TRUE	Concordant
1	29	BIR	64525	2542425322	345343463	
1	29	RIVM	64525	2542425322	345343463	
1	29	Matching?	TRUE	TRUE	TRUE	
2	5	BIR	5563'3	2232425322	013223250	
2	5	RIVM	5563'3	2232425322	013223250	
2	5	Matching?	TRUE	TRUE	TRUE	Concordant
2	30	BIR	5563'3	2232425322	013223250	
2	30	RIVM	5563'3	2232425322	013223250	
2	30	Matching?	TRUE	TRUE	TRUE	
3	13	BIR	31432	2232515323	234423372	
3	13	RIVM	31432	2232515323	234423372	
3	13	Matching?	TRUE	TRUE	TRUE	Concordant
3	25	BIR	31432	2232515323	234423372	
3	25	RIVM	31432	2232515323	234423372	
3	25	Matching?	TRUE	TRUE	TRUE	
4	17	BIR	22233	2422616321	444413282	
4	17	RIVM	22233	2422616321	444413282	
4	17	Matching?	TRUE	TRUE	TRUE	Concordant
4	23	BIR	22233	2422616321	444413282	
4	23	RIVM	22233	2422616321	444413282	
4	23	Matching?	TRUE	TRUE	TRUE	
5	14	BIR	32235	2642517332	842423364	
5	14	RIVM	32235	2642517332	842423364	Concordant
5	14	Matching?	TRUE	TRUE	TRUE	

Pair	DNA sample	Lab	ETR	MIRU	VNTR+	Concordant?
5	28	BIR	32235	2642517332	842423364	
5	28	RIVM	32235	2642517332	842423364	
5	28	Matching?	TRUE	TRUE	TRUE	
6	2	BIR	42436	2332517333	456443372	
6	2	RIVM	42436	2332517333	456443372	
6	2	Matching?	TRUE	TRUE	TRUE	Concordant
6	24	BIR	42436	2332517333	456443372	
6	24	RIVM	42436	2332517333	456443372	
6	24	Matching?	TRUE	TRUE	TRUE	
7	4	BIR	3344'3	2322613321	225423552	
7	4	RIVM	3344'3	2322613321	225423552	
7	4	Matching?	TRUE	TRUE	TRUE	Concordant
7	26	BIR	3344'3	2322613321	225423552	
7	26	RIVM	3344'3	2322613321	225423552	
7	26	Matching?	TRUE	TRUE	TRUE	
8	7	BIR	64466	24321022333	2-3313661	
8	7	RIVM	64466	24321022333	2d3313661	
8	7	Matching?	TRUE	TRUE	-	Discordant
8	22	BIR	64466	24321022333	223313661	
8	22	RIVM	64466	24321022333	2d3313661	
8	22	Matching?	TRUE	TRUE	FALSE	
9	11	BIR	54466	24321122333	383313461	
9	11	RIVM	54466	24321122333	383313461	
9	11	Matching?	TRUE	TRUE	TRUE	Concordant
9	21	BIR	54466	24321122333	383313461	
9	21	RIVM	54466	24321122333	383313461	
9	21	Matching?	TRUE	TRUE	TRUE	
10	6	BIR	83571	2731422222	337324373	Concordant
10	6	RIVM	83571	2731422222	337324373	

Pair	DNA sample	Lab	ETR	MIRU	VNTR+	Concordant?
10	6	Matching?	TRUE	TRUE	TRUE	
10	27	BIR	83571	2731422222	337324373	
10	27	RIVM	83571	2731422222	337324373	
10	27	Matching?	TRUE	TRUE	TRUE	
