# Supplementary material for: A Quantitative Evaluation of MIRU-VNTR Typing Against Whole-Genome Sequencing for Identifying Mycobacterium tuberculosis Transmission: A Prospective Observational Cohort Study
Source: eBioMedicine. 2018 Aug 1;34:122–30. doi: 10.1016/j.ebiom.2018.07.019 (PMC6116353; doi:10.1016/j.ebiom.2018.07.019)
Supplement: Supplementary file 3 — Supplementary material 3 [file mmc3.pptx]

## Slide 1
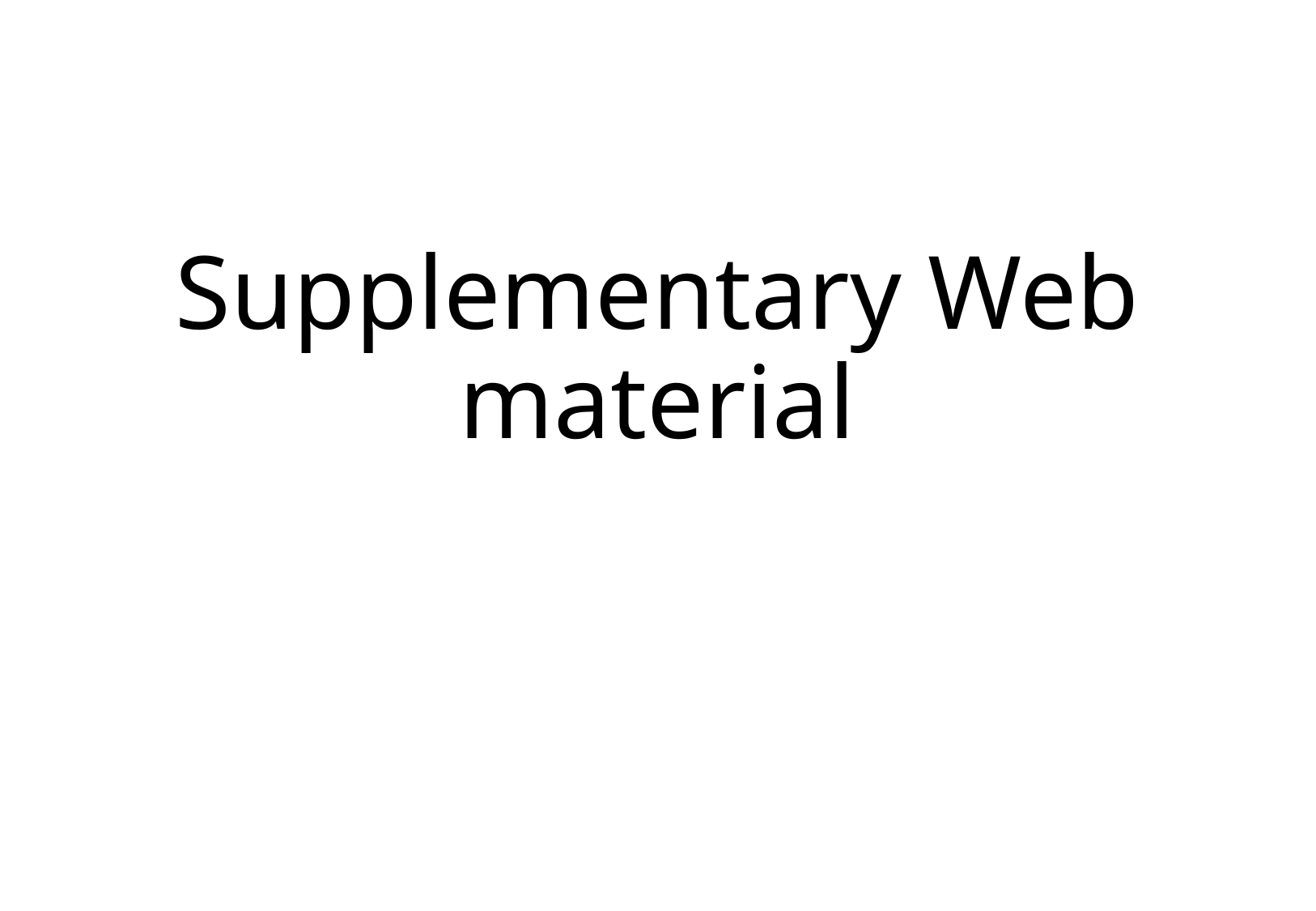

# Supplementary Web material

## Slide 2
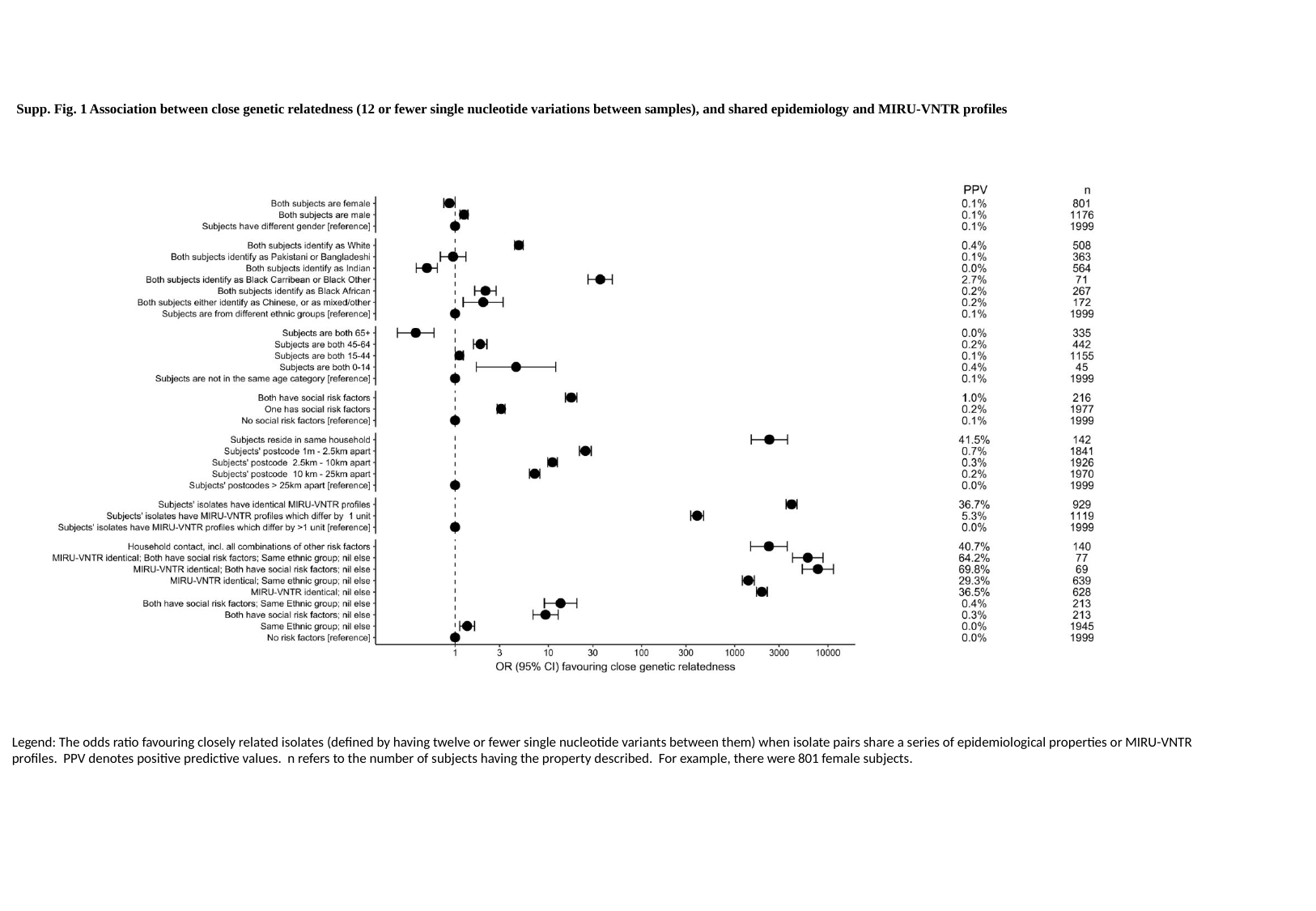

Supp. Fig. 1 Association between close genetic relatedness (12 or fewer single nucleotide variations between samples), and shared epidemiology and MIRU-VNTR profiles
Legend: The odds ratio favouring closely related isolates (defined by having twelve or fewer single nucleotide variants between them) when isolate pairs share a series of epidemiological properties or MIRU-VNTR profiles. PPV denotes positive predictive values. n refers to the number of subjects having the property described. For example, there were 801 female subjects.

## Slide 3
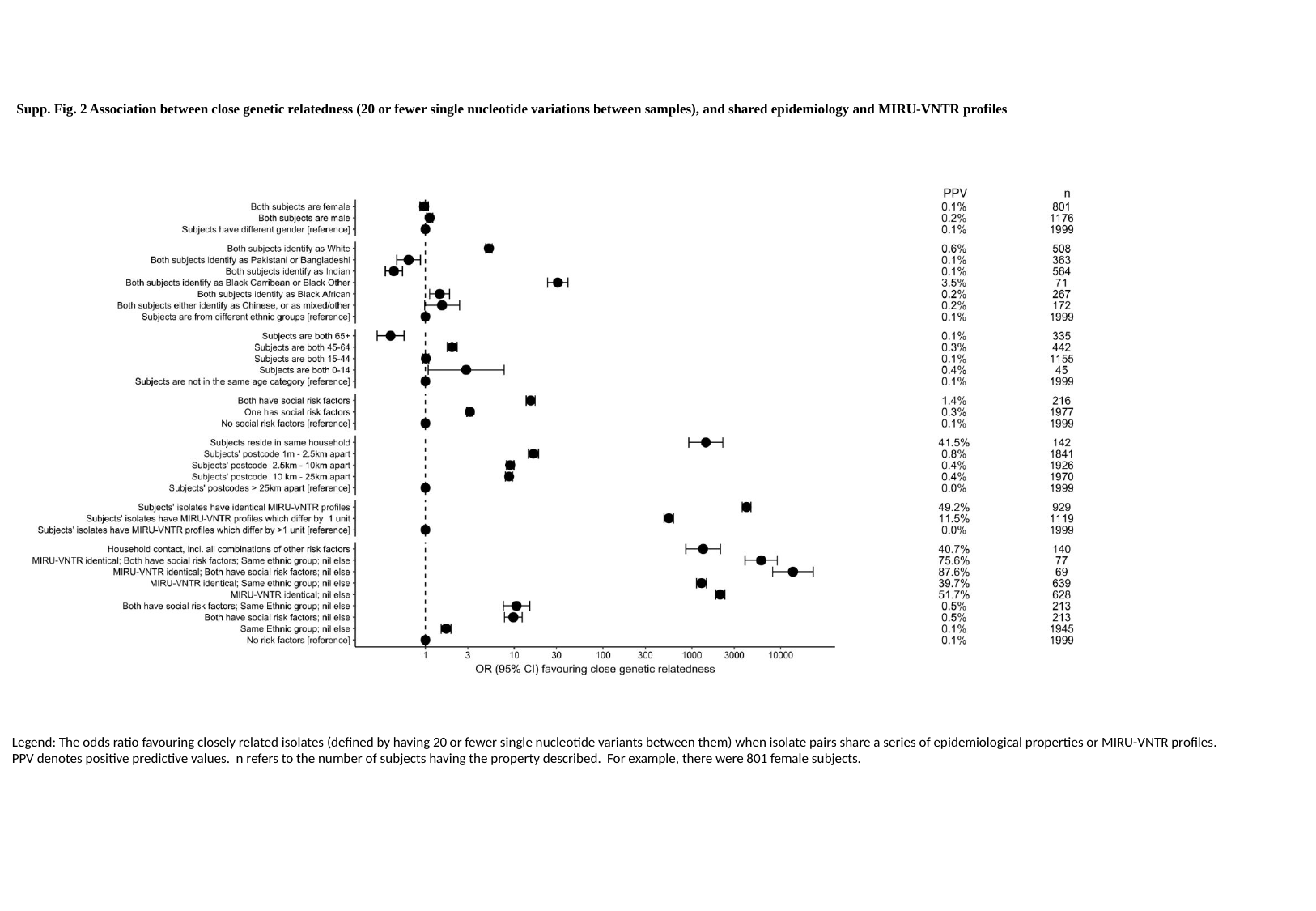

Supp. Fig. 2 Association between close genetic relatedness (20 or fewer single nucleotide variations between samples), and shared epidemiology and MIRU-VNTR profiles
Legend: The odds ratio favouring closely related isolates (defined by having 20 or fewer single nucleotide variants between them) when isolate pairs share a series of epidemiological properties or MIRU-VNTR profiles. PPV denotes positive predictive values. n refers to the number of subjects having the property described. For example, there were 801 female subjects.

## Slide 4
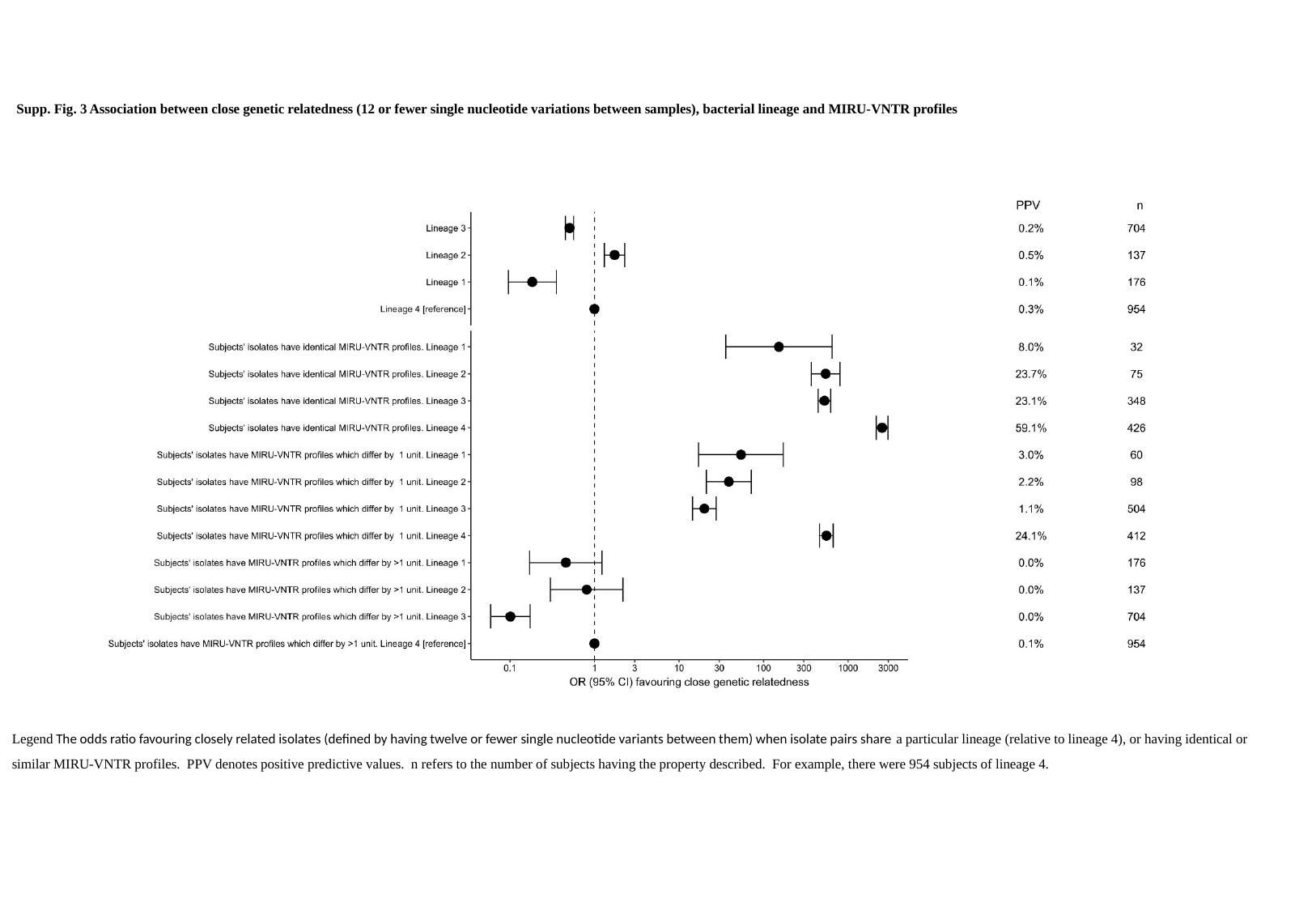

Supp. Fig. 3 Association between close genetic relatedness (12 or fewer single nucleotide variations between samples), bacterial lineage and MIRU-VNTR profiles
Legend The odds ratio favouring closely related isolates (defined by having twelve or fewer single nucleotide variants between them) when isolate pairs share a particular lineage (relative to lineage 4), or having identical or similar MIRU-VNTR profiles. PPV denotes positive predictive values. n refers to the number of subjects having the property described. For example, there were 954 subjects of lineage 4.

## Slide 5
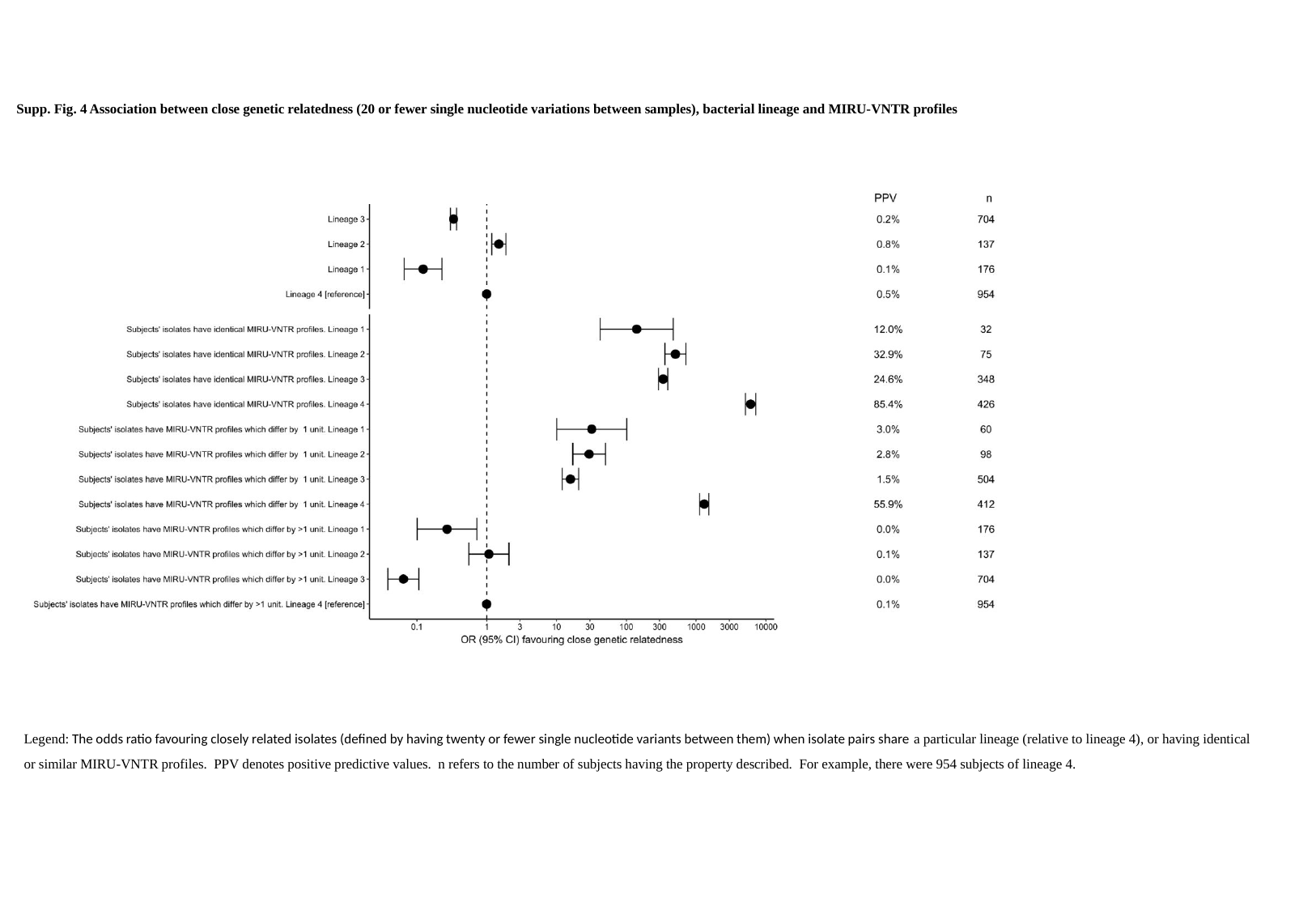

Supp. Fig. 4 Association between close genetic relatedness (20 or fewer single nucleotide variations between samples), bacterial lineage and MIRU-VNTR profiles
Legend: The odds ratio favouring closely related isolates (defined by having twenty or fewer single nucleotide variants between them) when isolate pairs share a particular lineage (relative to lineage 4), or having identical or similar MIRU-VNTR profiles. PPV denotes positive predictive values. n refers to the number of subjects having the property described. For example, there were 954 subjects of lineage 4.

## Slide 6
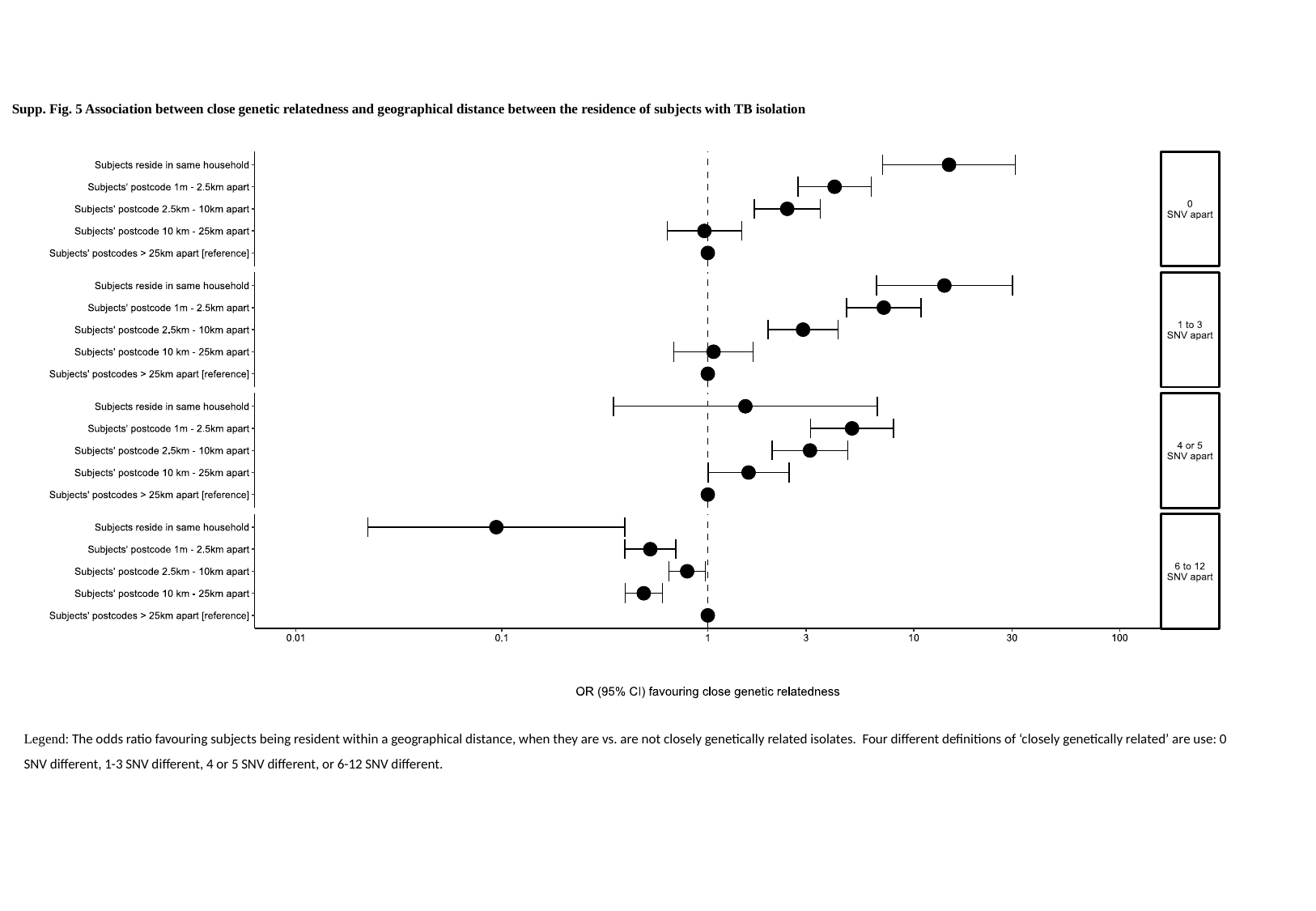

Supp. Fig. 5 Association between close genetic relatedness and geographical distance between the residence of subjects with TB isolation
Legend: The odds ratio favouring subjects being resident within a geographical distance, when they are vs. are not closely genetically related isolates. Four different definitions of ‘closely genetically related’ are use: 0 SNV different, 1-3 SNV different, 4 or 5 SNV different, or 6-12 SNV different.
